# Hypopigmented burn hypertrophic scar contains melanocytes that can be signaled to re-pigment by synthetic alpha-melanocyte stimulating hormone *in vitro*

**DOI:** 10.1371/journal.pone.0248985

**Published:** 2021-03-25

**Authors:** Bonnie C. Carney, Taryn E. Travis, Lauren T. Moffatt, Laura S. Johnson, Melissa M. McLawhorn, Cynthia M. Simbulan-Rosenthal, Dean S. Rosenthal, Jeffrey W. Shupp

**Affiliations:** 1 Department of Biochemistry and Molecular and Cellular Biology, Georgetown University School of Medicine, Washington, DC, United States of America; 2 Firefighters’ Burn and Surgical Research Laboratory, MedStar Health Research Institute, Washington, DC, United States of America; 3 Department of Surgery, Georgetown University School of Medicine, Washington, DC, United States of America; 4 Department of Surgery, The Burn Center, MedStar Washington Hospital Center, Washington, DC, United States of America; Boston University School of Medicine, UNITED STATES

## Abstract

There are limited treatments for dyschromia in burn hypertrophic scars (HTSs). Initial work in Duroc pig models showed that regions of scar that are light or dark have equal numbers of melanocytes. This study aims to confirm melanocyte presence in regions of hypo- and hyper-pigmentation in an animal model and patient samples. In a Duroc pig model, melanocyte presence was confirmed using *en face* staining. Patients with dyschromic HTSs had demographic, injury details, and melanin indices collected. Punch biopsies were taken of regions of hyper-, hypo-, or normally pigmented scar and skin. Biopsies were processed to obtain epidermal sheets (ESs). A subset of ESs were *en face* stained with melanocyte marker, S100β. Melanocytes were isolated from a different subset. Melanocytes were treated with NDP α-MSH, a pigmentation stimulator. mRNA was isolated from cells, and was used to evaluate gene expression of melanin-synthetic genes. In patient and pig scars, regions of hyper-, hypo-, and normal pigmentation had significantly different melanin indices. S100β *en face* staining showed that regions of hyper- and hypo-pigmentation contained the same number of melanocytes, but these cells had different dendricity/activity. Treatment of hypo-pigmented melanocytes with NDP α-MSH produced melanin by microscopy. Melanin-synthetic genes were upregulated in treated cells over controls. While traditionally it may be thought that hypopigmented regions of burn HTS display this phenotype because of the absence of pigment-producing cells, these data show that inactive melanocytes are present in these scar regions. By treating with a pigment stimulator, cells can be induced to re-pigment.

## 1 Introduction

Hypertrophic scars (HTSs) are common in patients after burn injury and have a continuum of pathophysiology that creates lasting morbidity. Patient populations that have baseline dark skin pigmentation are at a higher risk for development of HTS in general [1,2]. Additionally, dyspigmentation, specifically hypopigmentation in these patients is more noticeable and disfiguring. Prevention strategies that are used in the treatment of other aspects of HTS, such as compression therapy for scar thickness and pliability, have not been developed or utilized in preventing dyspigmentation [3]. Likewise, treatment strategies for these lesions are limited because the molecular and cellular etiology of dyschromia in HTS is unknown.

It is not an unreasonable *a priori* assumption that hypopigmented regions of HTS are the result of a paucity of melanocytes with the opposite being true for hyperpigmented regions. In an animal model of HTS we have demonstrated that dyschromia persists regardless of melanocyte number suggesting that hypo- or hyper-activity (not absence) of this cell type may be the etiologic factor in HTS pigmentation diathesis [4].

**Fig 1:**
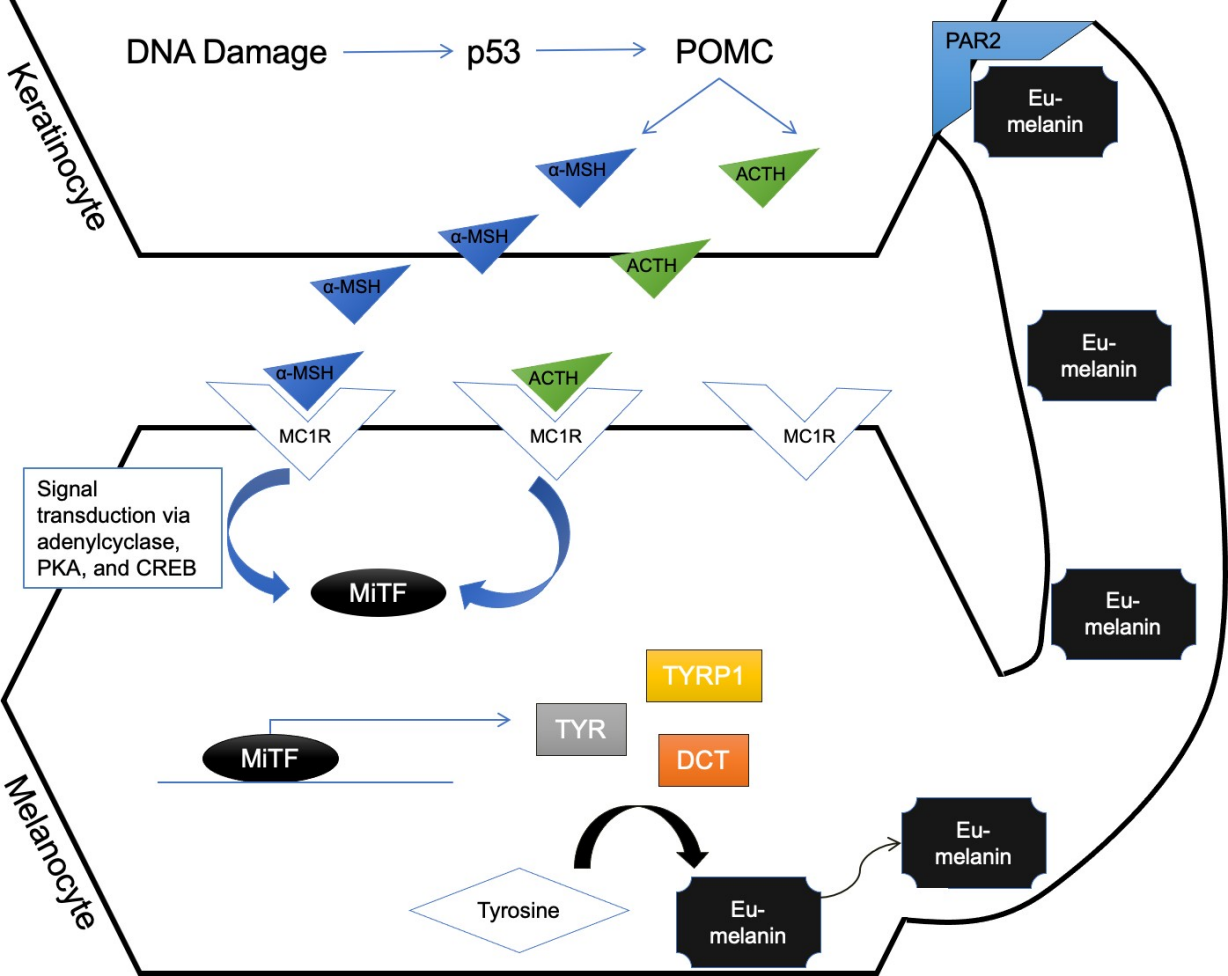
Melanin biosynthetic pathway. In the canonical pathway, keratinocytes that acquire DNA damage, usually through ultraviolet light (UVB), respond by upregulating tumor protein 53 (p53). p53 in turn is a transcription factor for proopiomelanocortin (POMC), thus upregulating its expression. POMC is proteolytically cleaved to its products, alpha melanocyte stimulating hormone (α-MSH) and adrenocorticotropin hormone (ACTH), which are secreted by keratinocytes and bind the melanocortin 1 (MC1R) on melanocyte membranes. This ligand-receptor binding propagates the signal transduction in melanocytes *via* adenylcyclase, protein kinase A (PKA), and cyclic AMP response element binding protein (CREB) to ultimately upregulate MiTF expression. When MiTF is phosphorylated through other mechanisms, it is translocated to the nucleus where it acts as a transcription factor for tyrosinase (TYR), tyrosinase related protein 1 (TYRP1), and dopachrome tautomerase (DCT), which are all enzymes required to convert tyrosine to eumelanin. Eumelanin is then packaged and transferred back to keratinocytes *via* protease-activated receptor 2 (PAR2) to protect against further keratinocyte nuclear damage.

While mechanisms for HTS dyschromia are unknown, cutaneous pigmentation homeostasis in uninjured tissue has been well characterized. Two cell types are involved in the synthesis of melanin: keratinocytes, which propagate the damage-associated signal, and melanocytes, which receive the signal and respond through signal transduction to synthesize eumelanin [5,6]. The illustrated pathway (Fig 1) is the canonical pathway by which skin cells make pigment; however, it is important to note that there are additional pathways that contribute to pigment synthesis, including through nerve growth factor receptor p75 (NGFR p75), prostaglandin E2 receptor 1 (EP1), endothelin B receptor (ETBR), granulocyte-macrophage colony stimulating factor (GM-CSFR), fibroblast growth factor 1 or 2 (FGFR1/2), and stem cell factor receptor (c-KIT), as well as some others [5,7,8].

Previous work by our group examined the melanin canonical biosynthetic pathway using a red Duroc pig model of HTS in an attempt to elucidate mechanisms of dyspigmentation [4]. Dysregulation of many pigmentation-specific key molecules was revealed, and the modulation of these molecules may be able to be used to target treatment of hyperpigmentation in the future.

In addition, we have shown through immunofluorescent staining for melanocyte marker S100 calcium binding protein B (S100β), qRT-PCR, and primary *in vitro* cell culture that melanocytes are present in hypopigmented Duroc pig scar [4,9]. While the use of Duroc pigs is an accepted animal model for HTS, adapting it for the study of dyschromia is novel. Confirmation of dyschromia-related similarities between porcine and human lesions is essential moving forward to assess model translatability. If the presence of melanocytes in hypopigmented burn scars in patients can be confirmed, the use of Duroc pig dyschromic scars will be useful in developing treatments in future work.

The overall goal of studying dyschromia in HTS is to develop treatments that are mechanistic in nature and may be tissue sparing or alleviate the need for surgery. The canonical pigmentation signaling cascade was therefore leveraged when a pigmentation stimulator was chosen. Norleucine D-Phenylalanine (NDP) α-MSH, a synthetic form of α-MSH, has been used *in vitro* and *in vivo* to study the initiation of pigmentation in non-burn dyschromia. As discussed in a recent review, this compound has been used in clinical trials to induce tanning in healthy volunteers, in patients with erythropoietic protoporphyria (EPP), and in patients with vitiligo [10]. In this set of experiments, the hypothesis that supplying hypo-pigmented cells with α-MSH or NDP α-MSH could lead to re-pigmentation was tested. In this pathway, α-MSH is secreted from keratinocytes, binds to MC1R, and the ligand/receptor binding initiates signal transduction in the melanocyte to start gene transcription of the 3 genes required for converting tyrosine to eu-melanin (TYR, TYRP1, and DCT) (Fig 1). This treatment was chosen based on data that shows that POMC (the parent molecule of α-MSH) signaling is absent in hypopigmented scar and likely requires exogenous replacement [4,11]. This hypothesis was tested in primarily-derived patient cells from hyper-, hypo-, and normally pigmented scar and skin. These cells were stimulated with α-MSH or NDP α-MSH and their response was evaluated.

## 2 Results

### 2.1 Pigs

#### 2.1.1 Full thickness skin injury can result in dyspigmented hypertrophic scar

**Fig 2:**
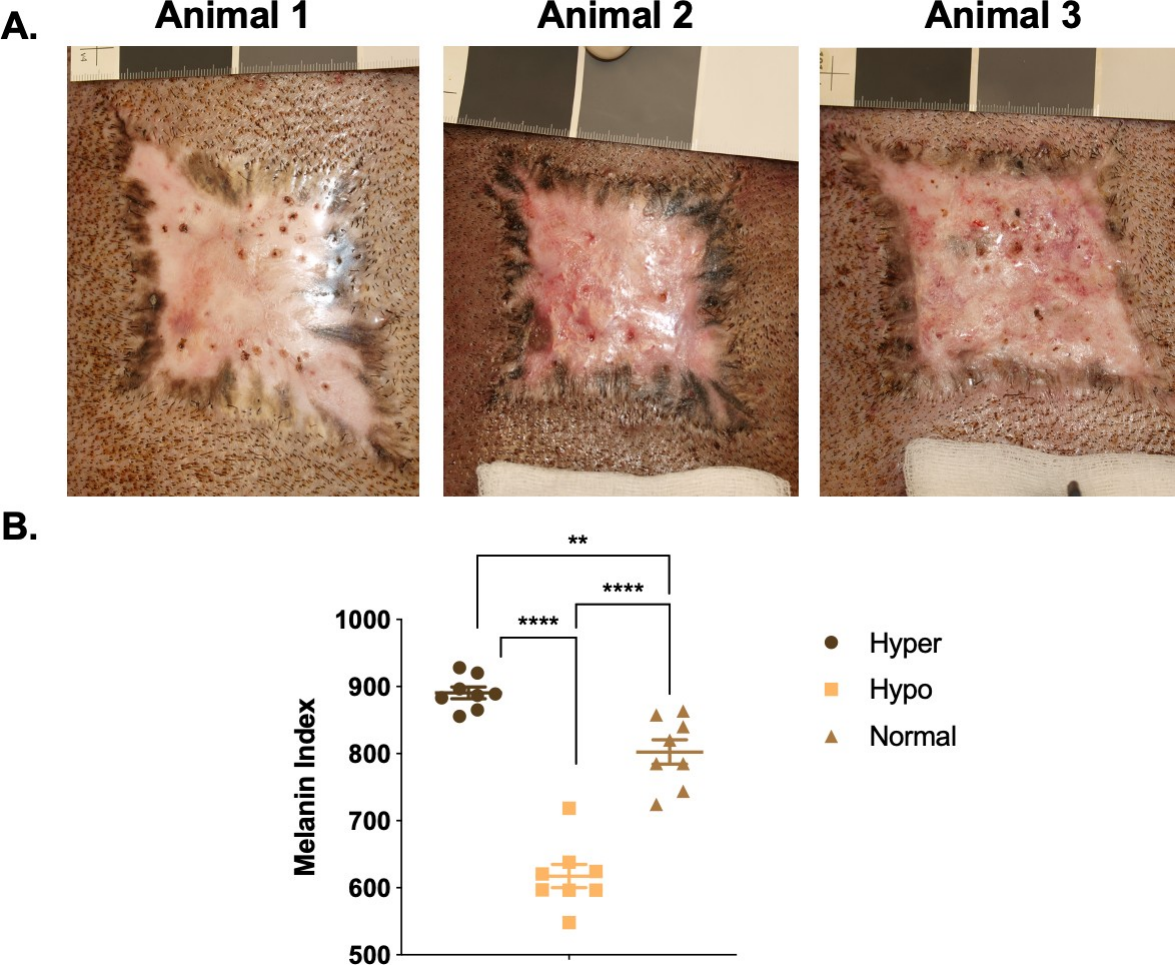
Heterogeneous dyschromia with hyper- and hypo-pigmentation develops after full thickness excision wounding in porcine HTS. Examples of HTS from 3 pigs (A). SCC non-invasive skin probe was used to measure melanin in the different pigmentation phenotypes (B) (mean ± SEM, n = 8 scars). **p<0.01, ****p<0.0001.

Hypertrophic scars in Duroc pigs are predominantly hyperpigmented on the periphery with hypopigmentation in the middle of the lesions (Fig 2A and Table 1). By SCC, there are significant differences between all 3 pigmentation phenotypes when melanin index is analyzed (p<0.01).

**Table 1:** Porcine demographics, injury details, and acute burn and hypertrophic scar management.

Animal #	Age at time of sampling (months)	Gender	Breed	Fitzpatrick skin type	Age of scar (months)	Mechanism of Injury	Location of dyspigmented scars	Acute burn management of specific scar area	Previous scar treatments
P1	6.3	M	Duroc	5	2	Full thickness excision	R. flank	D	None
P2	6.3	M	Duroc	5	2	Full thickness excision	R. flank	D	None
P3	6.3	M	Duroc	5	2	Full thickness excision	R. flank	D	None

For acute burn management, D = Graft failure and healing by secondary intention.

#### 2.1.2 Staining of pig-derived biopsies show the structural and microscopic pigmentation differences in the epidermis between different phenotypes

**Fig 3:**
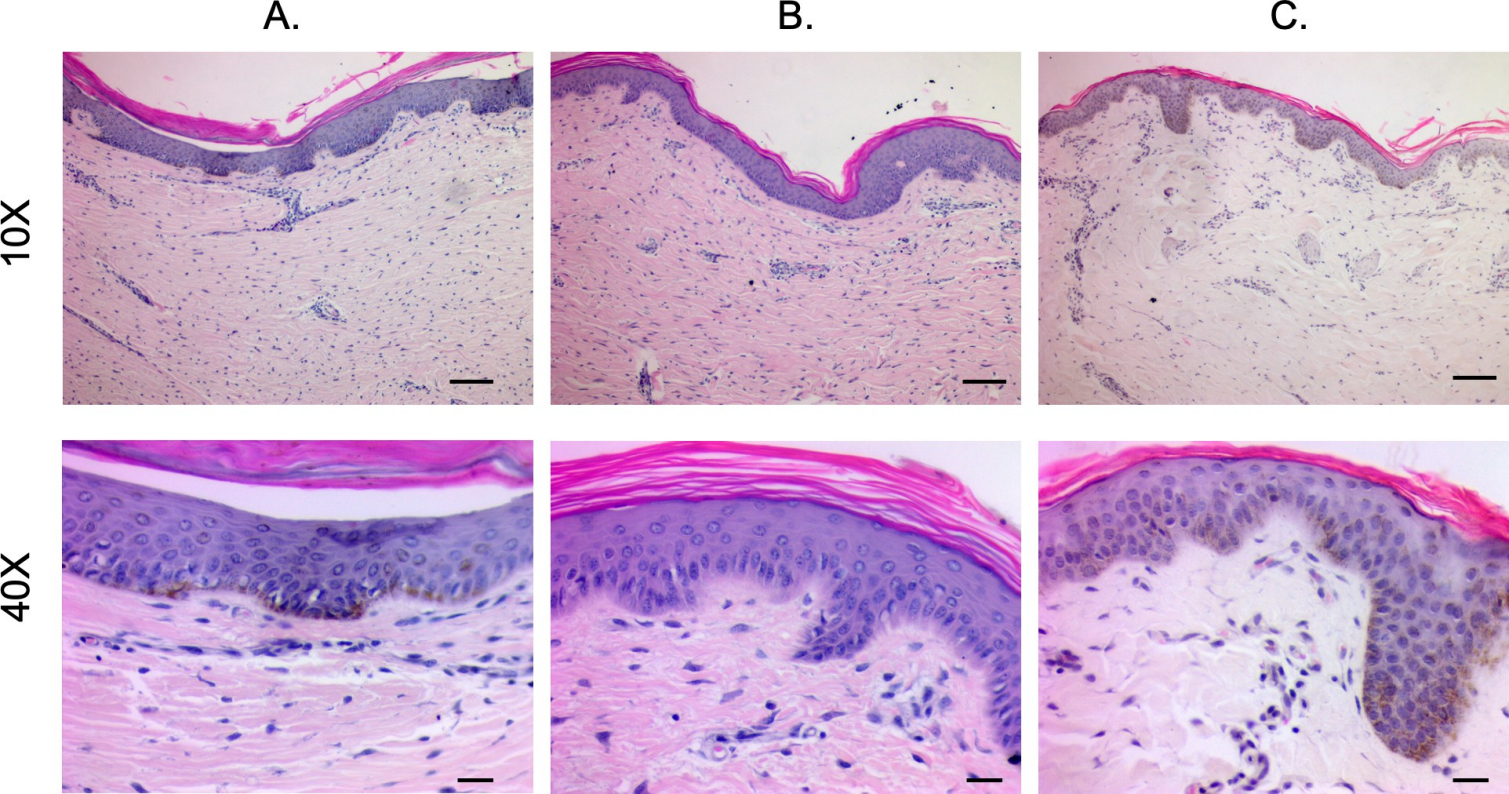
H&E staining reveals structural architecture of hyper- and hypo-pigmented porcine HTS compared to normal skin. Punch biopsies of distinct regions of hyper- (A), hypo- (B), and normally-pigmented (C) scar and skin were taken and were FFPE and H&E stained. (Scale bar = 100 μm for 10X, top and 20 μm for 40X, bottom).

**Fig 4:**
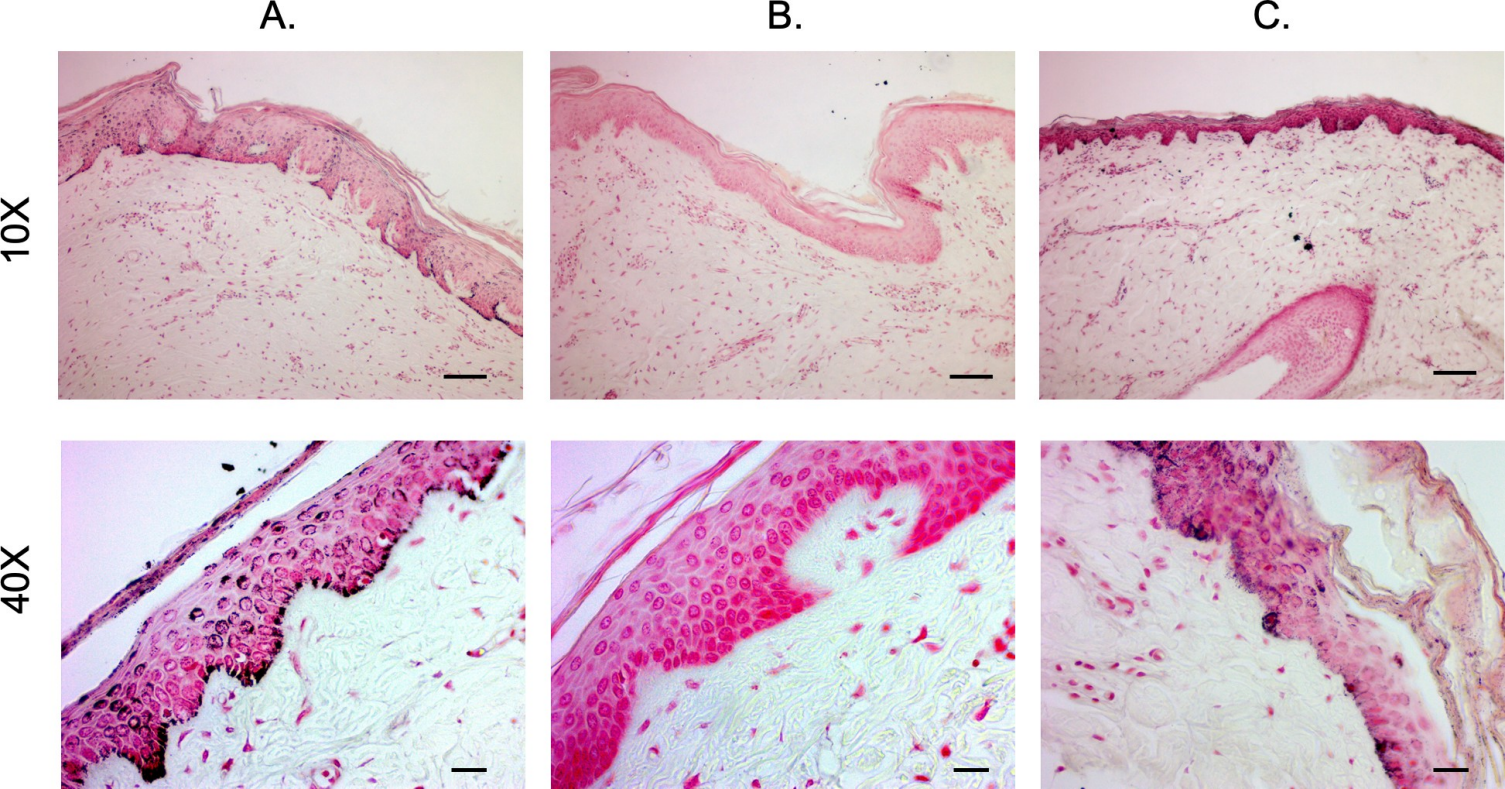
Fontana-Masson staining reveals melanin deposition of hyper- and hypo-pigmented porcine HTS compared to normal skin. Punch biopsies of distinct regions of hyper- (A), hypo- (B), and normally-pigmented (C) scar and skin were taken and were FFPE and Fontana-Masson stained. (Scale bar = 100 μm for 10X, top and 20 μm for 40X, bottom).

H&E-stained sections of hyper-, hypo-, and normally pigmented scar and skin of pig-derived biopsies revealed that hyper- and hypo-pigmented scar had rete ridge flattening, increased epidermal and dermal thickness, increased cellularity, increased vascularity, and a more disorganized collagen structure compared to normal skin (Figs 3 and S1). These features of scar have been quantitatively measured in the same pig samples in a recent report where we showed statistical increases in the above mentioned parameters [12]. These features identify them as being representative of the known histologic features of HTS. In all pigs, similar structural patterns were observed. Fontana-Masson staining confirmed that regions of hypopigmentation did not contain melanin (Fig 4). Regions of hyperpigmentation had diffuse melanin staining throughout all layers of the epidermis including the stratum corneum. This staining closely resembled melanin distribution in normally pigmented skin. In all patients, the same melanin patterns were observed in the different phenotypes.

#### 2.1.3 Regions of hyper- and hypo-pigmentation contain melanocytes in equal numbers by *en face* staining

**Fig 5:**
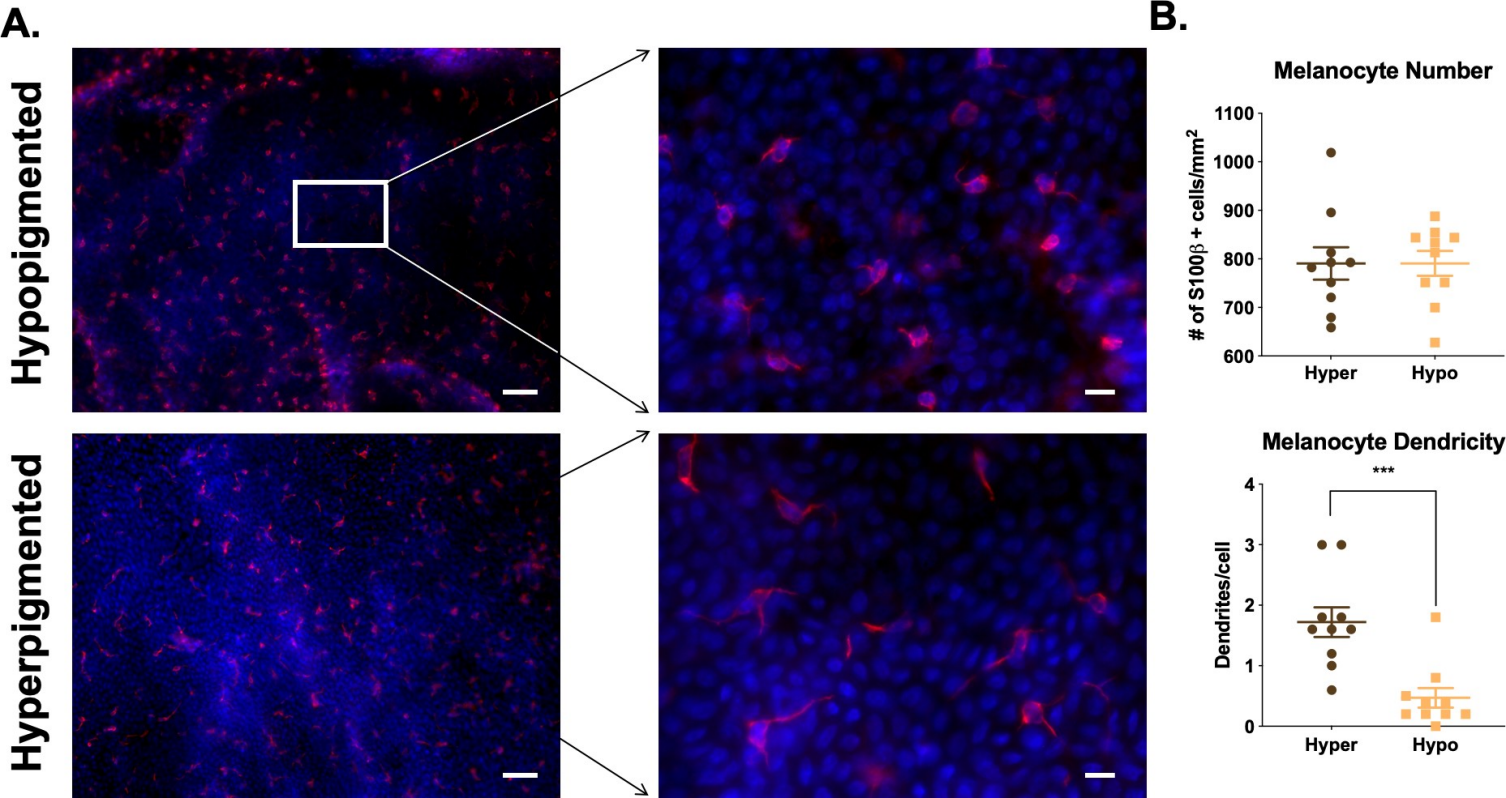
Regions of hyper- and hypo-pigmentation in pig HTS contain melanocytes in equal numbers. Epidermal sheets from regions of hyper- or hypo-pigmentation were stained for melanocyte marker, S100β by *en face* staining. S100β (red), DAPI (blue). Scale Bar = 50 μm at 10X (left) or 10 μm at 40X (right) (A). Melanocytes were counted in each region of pigmentation. Differences are not significant (B, top). Melanocyte dendrites were counted in each region of pigmentation (B, bottom). Images are from Pig #1 from Table 1. (mean ± SEM, n = 10 scars, ***p<0.001).

Melanocyte presence was further investigated with *en face* staining. Hyperpigmented scar areas contained 798.2 ± 36.2 melanocytes/mm^2^ and hypopigmented scar areas contained 787.3 ± 26.6 melanocytes/mm^2^ (n = 9 scars, p = 0.72, n.s.) (Fig 5). *En face* staining allows for the visualization of skin, looking directly down at the basal layer of the epidermis, as opposed to FFPE staining of skin where a cross section is viewed. The counterstaining with DAPI in the epidermal sheets shows the dermal papillae that are prominent in hyper- and hypo-pigmented scar, and shows how melanocytes are distributed in a cell density that is expected for melanocytes. Hyperpigmented regions had activated melanocytes with 1.78 ± 0.3 dendrites/cell, while hypopigmented regions had inactive melanocytes with 0.5 ± 0.2 dendrites/cell (n = 9, p = 0.0005) (Fig 5B).

### 2.2 Patients

#### 2.2.1 Full thickness skin injury can result in dyspigmented hypertrophic scar

**Fig 6:**
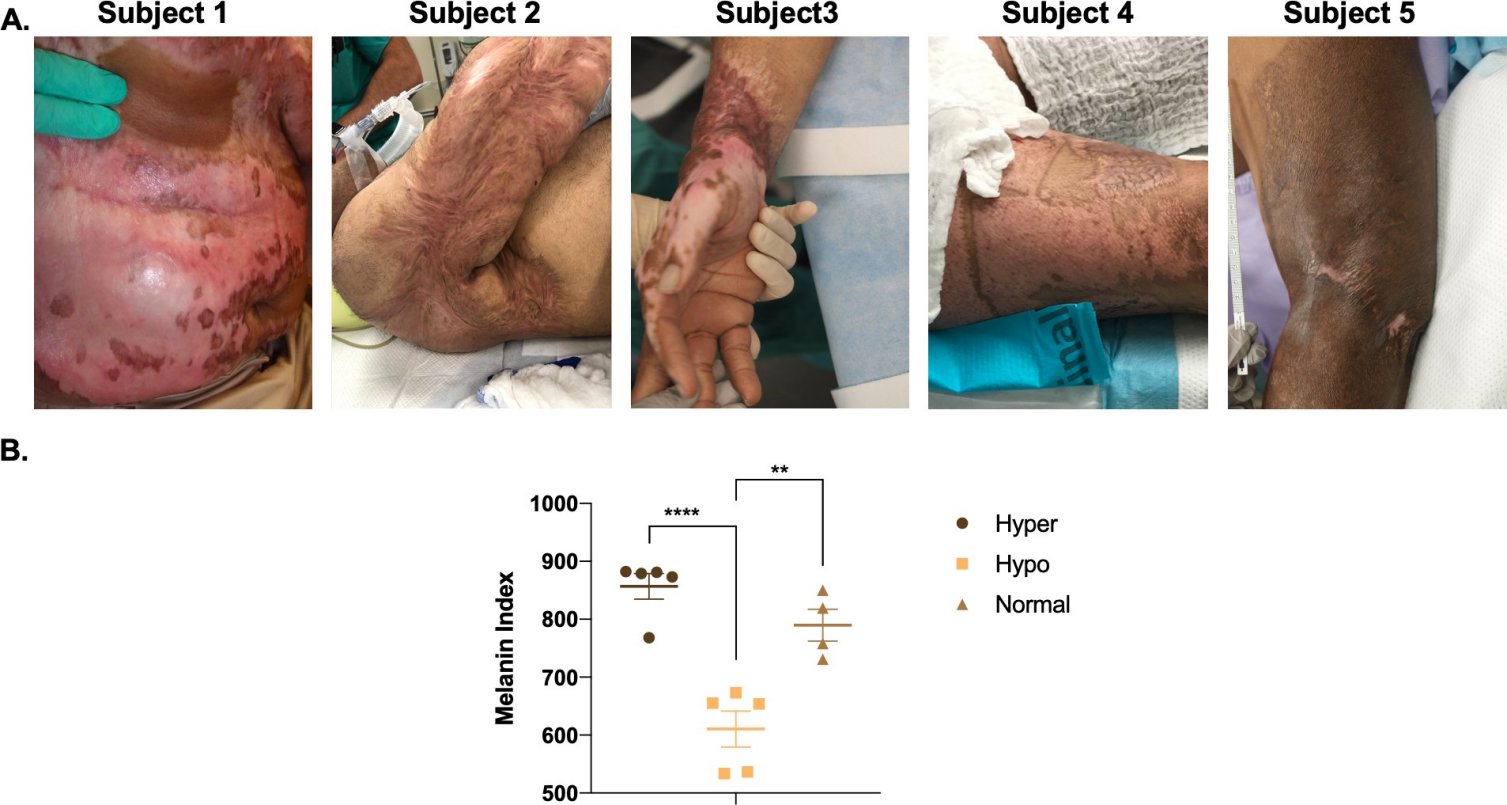
Heterogeneous dyschromia with hyper- and hypo-pigmentation develops after burn injury in patient HTS. Examples of HTS from Subjects 1–5 from Table 2 (A). SCC non-invasive skin probe was used to measure melanin in the different pigmentation phenotypes (B) (mean ± SEM, n = 5 scars). **p<0.01, ****p<0.0001.

Patient dyschromic scars are more variable compared to animal lesions due to the variable nature of their injuries (Fig 6A). Dyschromia develops on all areas of the body (Fig 6A and Table 2). By SCC, there are significant differences in melanin index in hyper- *vs*. hypo-pigmented scar and between hypopigmented scar and normally pigmented skin (p<0.01) (Fig 6B).

**Table 2:** Subject demographics, injury details, and acute burn and hypertrophic scar management.

Subject #	Age at time of sampling (years)	Gender	Race	Fitzpatrick skin type	Age of scar (months)	Mechanism of Injury	Location of dyspigmented scars	Acute burn management of specific scar area	Previous scar treatments
1	63	F	AA	5	15	Fire/flames	R. chest flank and axilla	A,B	1,2,3,4,5
2	35	M	A	3	395	Unknown	R. posterior axilla	Unknown	2,3,4
3	55	M	AA	5	19	Scald	R. wrist	E	1,2
4	37	M	H	3	11	Fire/flames	R. thigh	A,D	1,2
5	52	M	AA	5	15	Flash	L. elbow	B	1,2
6	22	M	H	4	20	Fire/flames	L. thigh	A, C	1,2
7	60	M	W	2	12	Volcanic ash	R. thigh	A	1,2
8	34	M	AA	6	6	Fire/flames	Bilateral hands	A (hyper), E (hypo)	1,2
9	52	F	AA	6	6	Fire/flames	R. thigh	A (hypo), F (hyper)	1,2
10	38	M	AA	5	8	Unknown	L. shoulder	A	1,2
11	52	M	AA	5	4	Electrical injury	R. thigh	F	--
12	39	F	AA	5	4	Fire/flames	L. thigh	C	1,2
12	54	M	H	4	6	Electro-thermal	L. arm	B	1
13	45	F	AA	6	63	Donor Site	R. thigh	F	--

For race, AA = African American, A = Asian, H = Hispanic, W = white. For acute burn management, A = Excision and autografting, B = Excision and xenografting, C = excision and allografting, D = Graft failure and healing by secondary intention, E = Wound care and healing by secondary intention, outside hospital, F = scar resulting from creation of donor site. For previous scar treatments, 1 = Custom compression garments, 2 = Fractional ablative CO2 laser scar revision with topical drug delivery of triamcinolone, 3 = Burn scar excision and graft placement, 4 = Burn scar release and local tissue rearrangement, 5 = Steroid injection.

#### 2.2.2 Staining of patient-derived biopsies show the structural and microscopic pigmentation differences in the epidermis between different phenotypes

**Fig 7:**
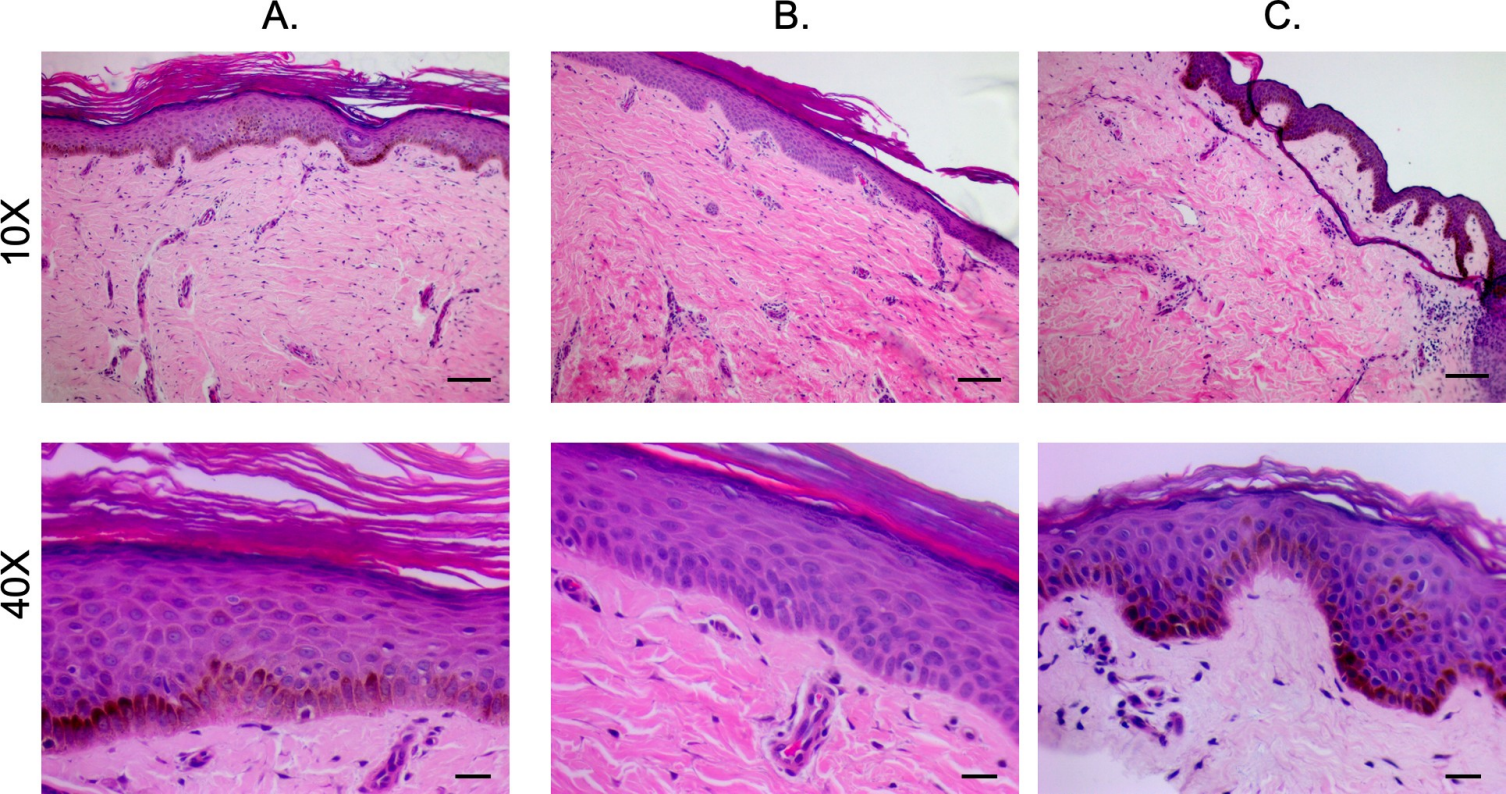
H&E staining reveals structural architecture of hyper- and hypo-pigmented patient HTS compared to normal skin. Punch biopsies of distinct regions of hyper- (A), hypo- (B), and normally-pigmented (C) scar and skin were taken and were FFPE and H&E stained. (Scale bar = 100 μm for 10X, top and 20 μm for 40X, bottom).

**Fig 8:**
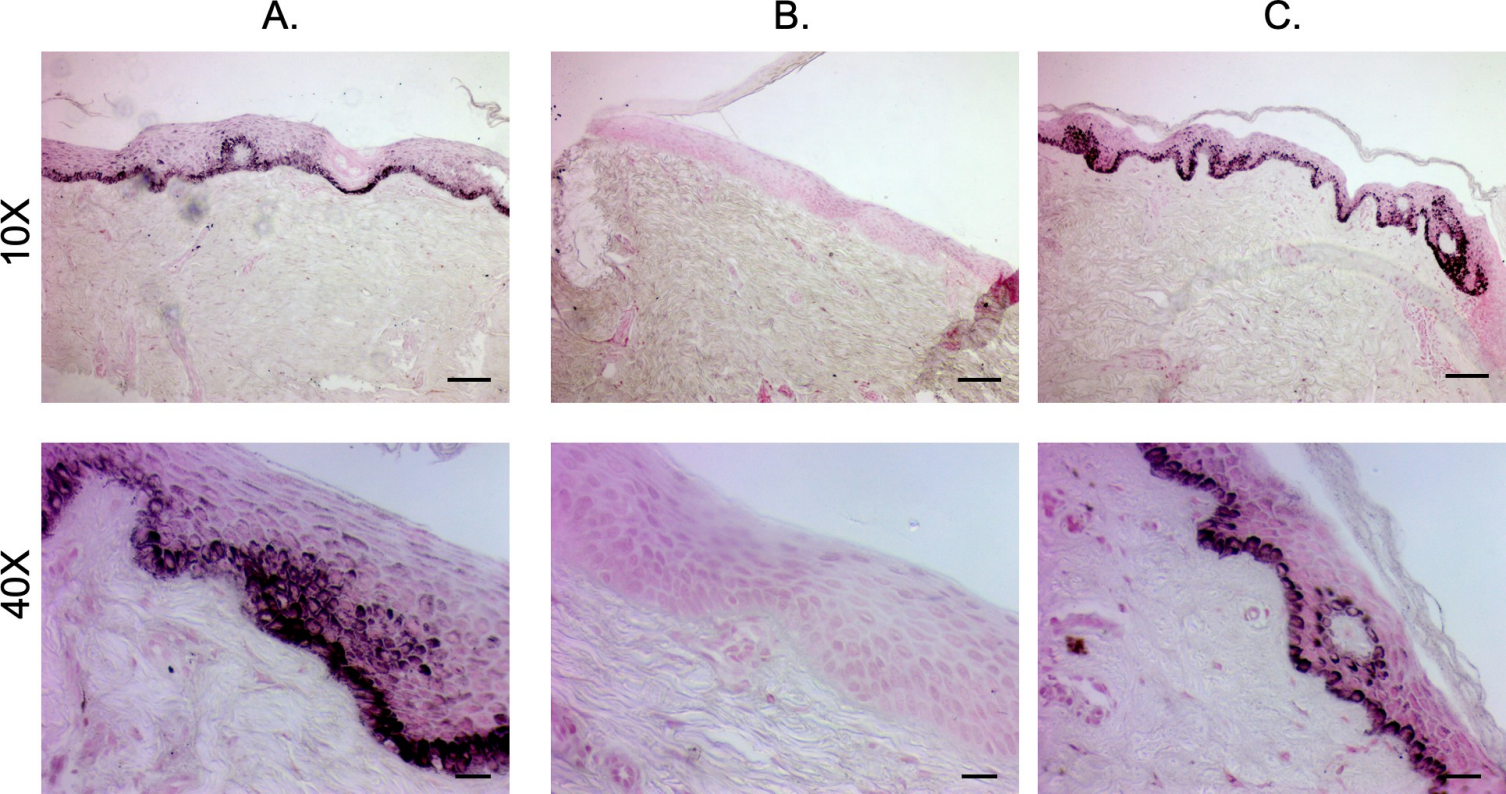
Fontana-Masson staining reveals melanin deposition of hyper- and hypo-pigmented patient HTS compared to normal skin. Punch biopsies of distinct regions of hyper- (A), hypo- (B), and normally-pigmented (C) scar and skin were taken and were FFPE and Fontana-Masson stained. (Scale bar = 100 μm for 10X, top and 20 μm for 40X, bottom).

The H&E staining of human patient samples concurs with the findings in the pig samples in confirming a number of known aspects of HTS (Fig 7). The Fontana-Masson staining also agreed with the pig samples, and importantly confirmed that the hypopigmented scar did not contain any melanin microscopically (Fig 8).

#### 2.2.3 Regions of hyper- and hypo-pigmentation contain melanocytes in equal numbers by *en face* staining

**Fig 9:**
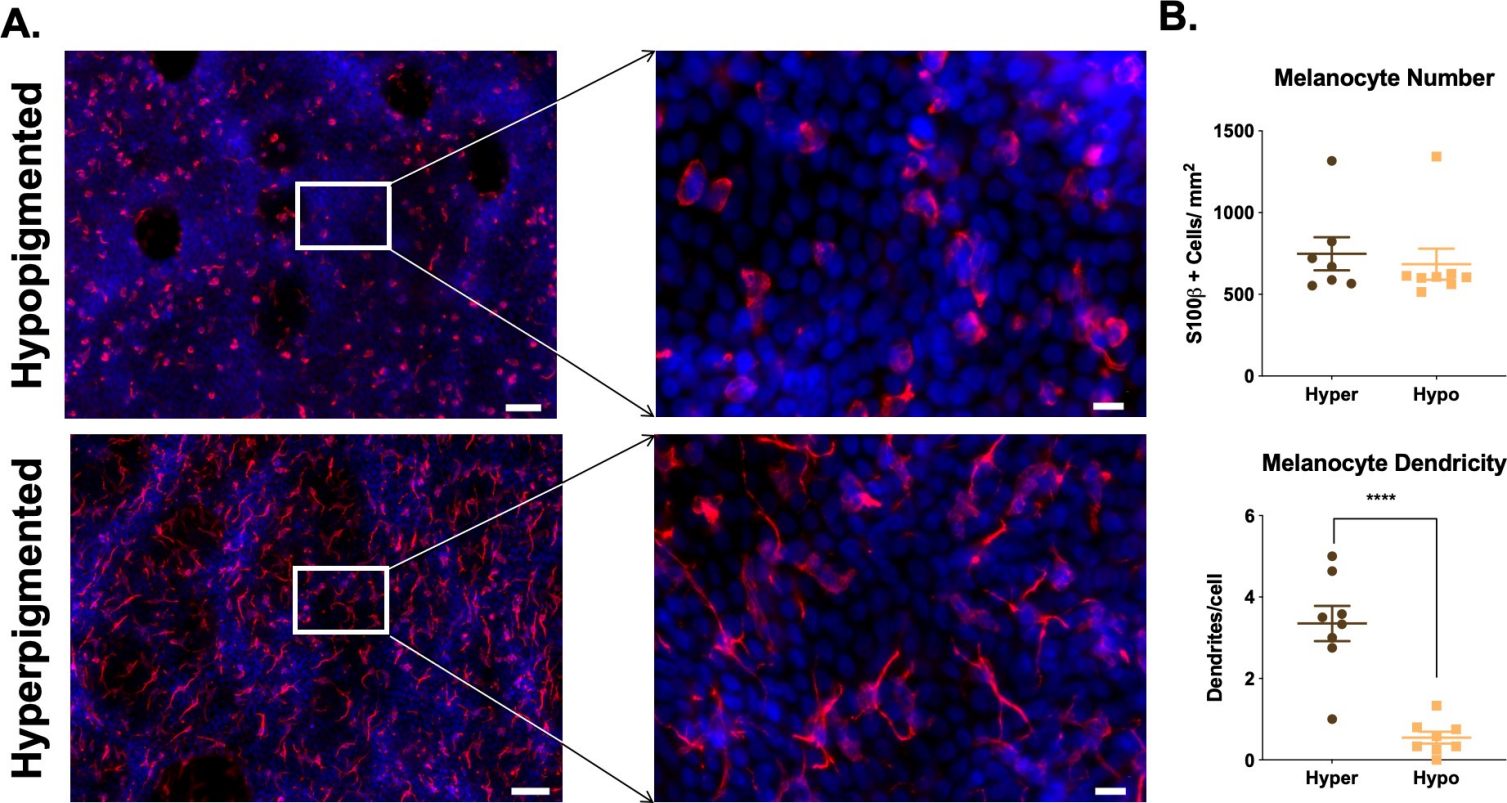
Regions of hyper- and hypo-pigmentation in patient HTS contain melanocytes in equal numbers. Epidermal sheets from regions of hyper- or hypo-pigmentation were stained for melanocyte marker, S100β by *en face* staining. S100β (red), DAPI (blue). Scale Bar = 50 μm at 10X (left) or 10 μm at 40X (right) (A). Melanocytes were counted in each region of pigmentation (B, top). Melanocyte dendrites were counted in each region of pigmentation (B, bottom). Images are from Patient #1 from Table 2. (Scale bar = 50 μm for 10X, top and 20 μm for 40X, bottom) (mean ± SEM, n = 8 scars, ***p<0.001).

Melanocytes were ubiquitous in regions of both hyper- and hypo-pigmentation, with 748.16 ± 137.28 cells/mm^2^ *vs*. 683.95 ± 95.02 cells/mm^2^ (n = 8, p = 0.6520), respectively (Fig 9A). Melanocytes are distributed in a cell density that is expected for skin, and is consistent with staining for melanocytes in porcine HTS. S100β-positive cells were identified in hypo- and hyper-pigmented scar in all patients (n = 8) (S2–S8 Figs). Hyperpigmented regions had activated melanocytes with 3.35 ± 0.71 dendrites/cell, while hypopigmented regions had inactive melanocytes with 0.55 ± 0.24 dendrites/cell (Fig 9B).

#### 2.2.4 Heterogeneous injury types result in heterogeneous dyschromic HTS with regions of hyper and hypo-pigmentation

**Fig 10:**
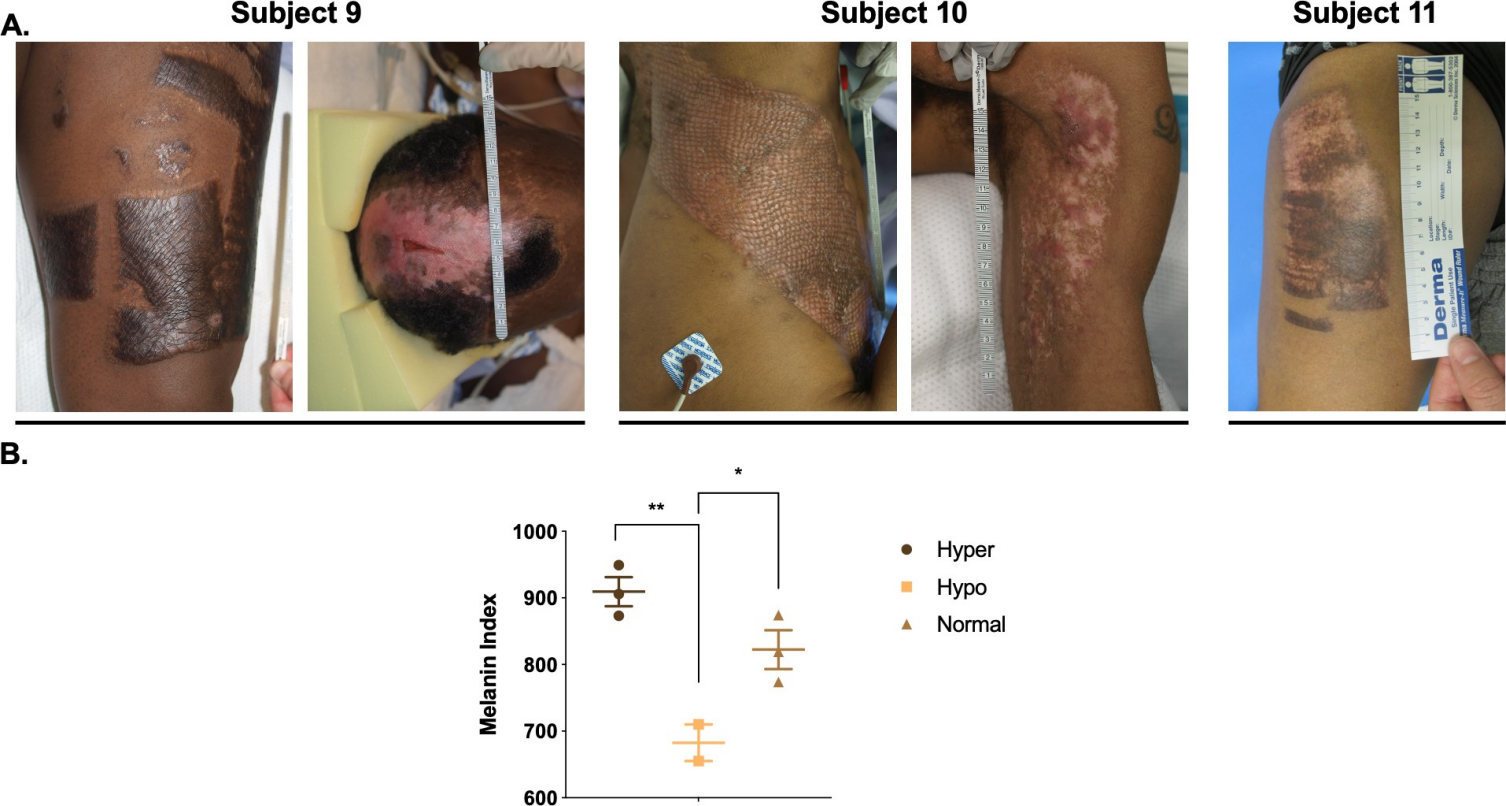
Heterogeneous dyschromia with hyper- and hypo-pigmentation develops after burn injury in patient HTS. Examples of HTS from Subjects 9–11 from Table 2 (A). SCC non-invasive skin probe was used to measure melanin in the different pigmentation phenotypes (B) (mean ± SEM, n = 3 scars). *p<0.05, **p<0.01.

**Fig 11:**
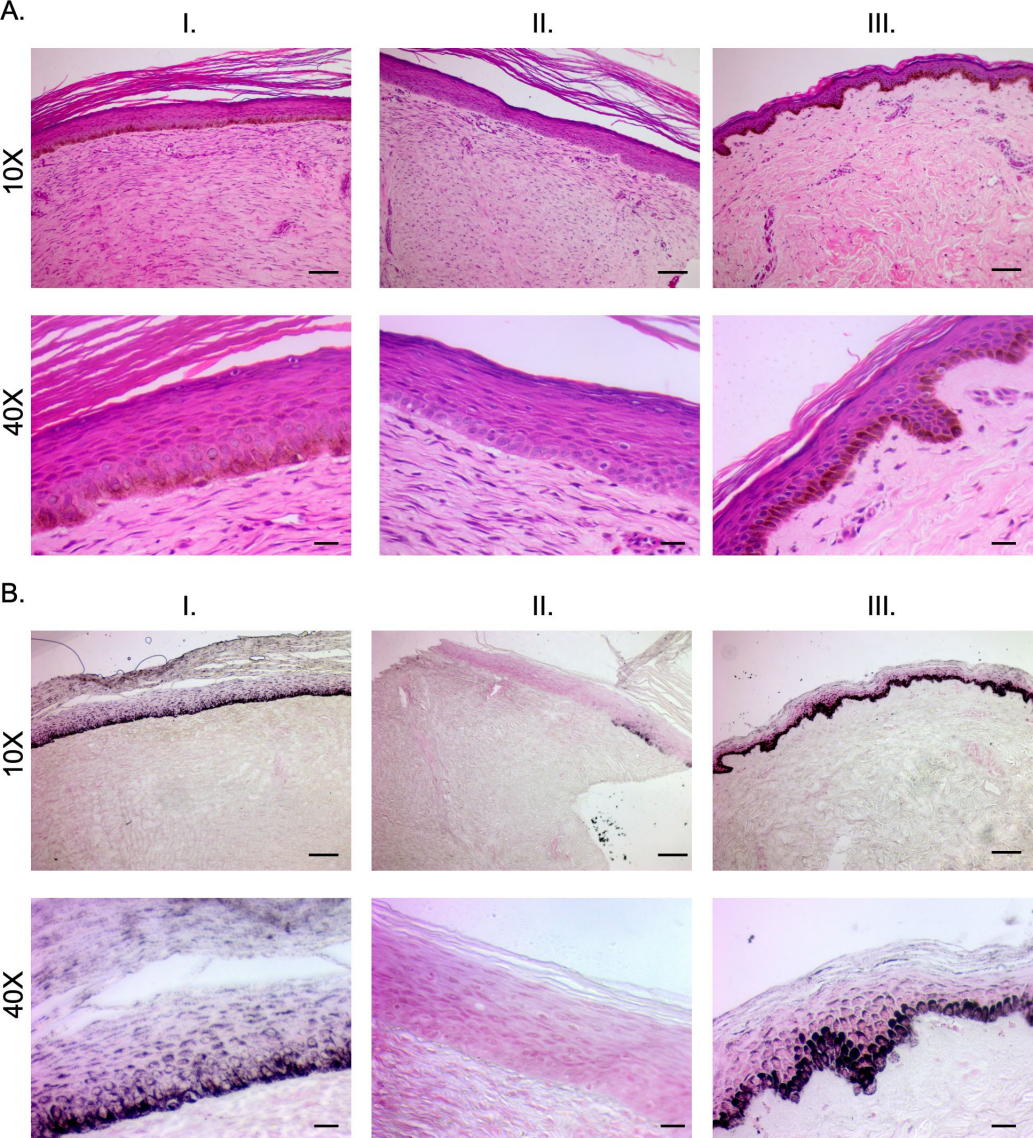
Staining reveals structural architecture and melanin deposition in hyper- and hypo-pigmented patient HTS compared to normal skin. Punch biopsies of distinct regions of hyper- (I), hypo- (II), and normally-pigmented (III) scar and skin were taken and were FFPE and H&E stained (A). The same biopsies were Fontana-Masson stained (B). Images are from Subject #6 in Table 2. (Scale bar = 100 μm for 10X, and 20 μm for 40X).

Five additional patients were enrolled and had photographs, non-invasive skin probe SCC measurements of melanin, and tissue biopsies collected. The scar dyschromia was variable depending on the initial injury (Fig 10A). By SCC, there are significant differences in melanin index in hyper- *vs*. hypo-pigmented scar and between hypopigmented scar and normally pigmented skin (p<0.05) (Fig 10B). H&E (Fig 11A) and Fontana-Masson (Fig 11B) staining was used to characterize scar architecture and confirm the absence of melanin-producing melanocytes from hypo-pigmented regions of scar.

#### 2.2.5 Regions of hyper- and hypo-pigmentation contain melanocytes by primary cell culture in patient samples

**Fig 12:**
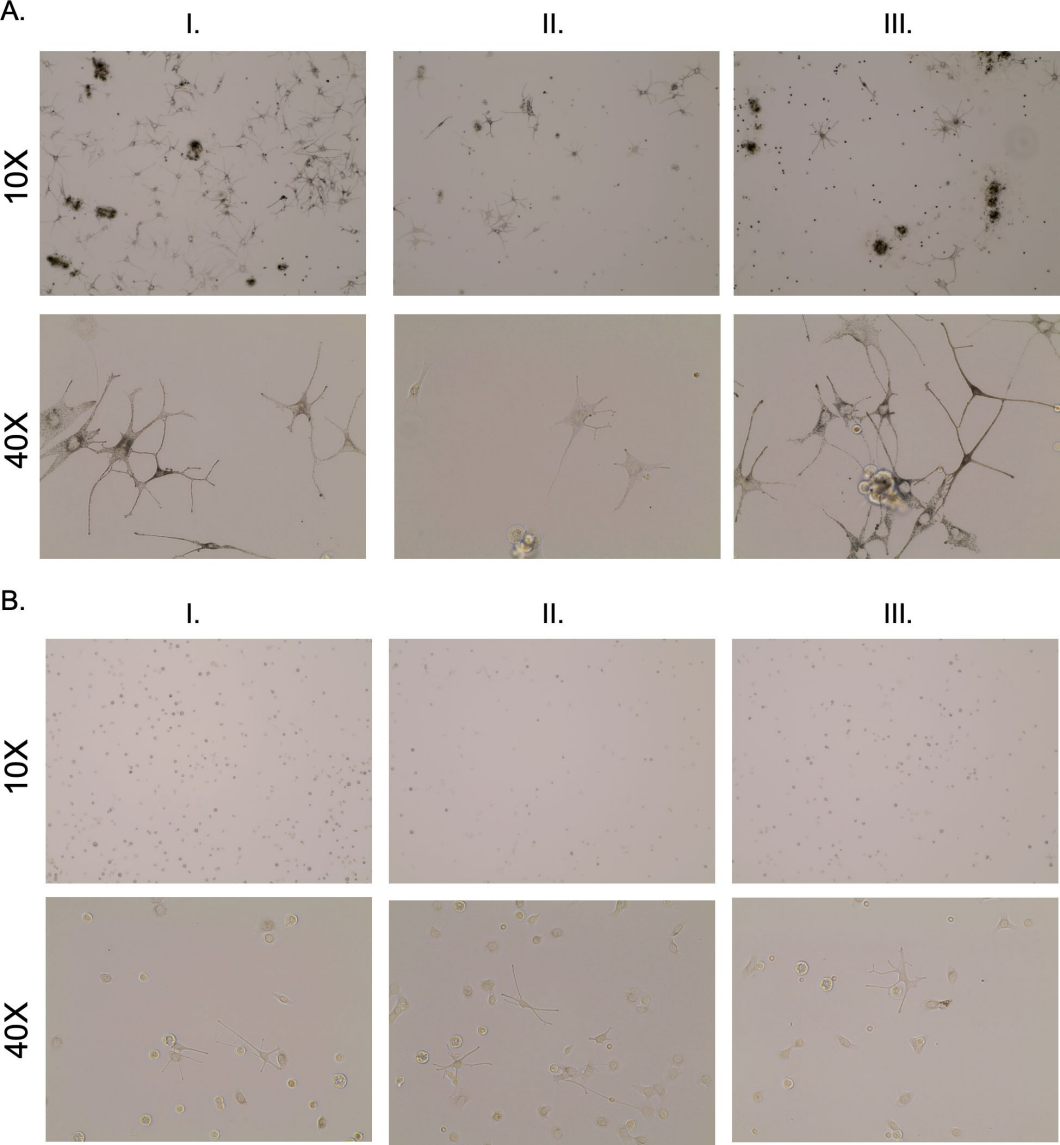
Melanocytes were cultured from biopsies regardless of pigmentation phenotype. The sample biopsies were treated with dispase to isolate epidermal cells which were seeded in culture. Images were taken under bright field microscopy at a fixed light intensity after 3 days in culture. Images are from Subject #9 (A) and 12 (B) in Table 2.

In each of the 5 hypopigmented scar samples, melanocytes were isolated and grown in culture (Figs 12 and S9–S14). These melanocytes were hypopigmented by brightfield microscopy compared to the melanocytes isolated from the normally pigmented skin and hyper-pigmented scar. When hyper- and hypo-pigmented epidermal cells were seeded into culture, both pigmentation types resulted in cells that have characteristic melanocyte morphology. The hypopigmented melanocytes do not retain their “inactivated” phenotype observed in *en face* staining when put into culture.

#### 2.2.6 Hypopigmented melanocytes can be stimulated to produce melanin in media containing α-MSH

**Fig 13:**
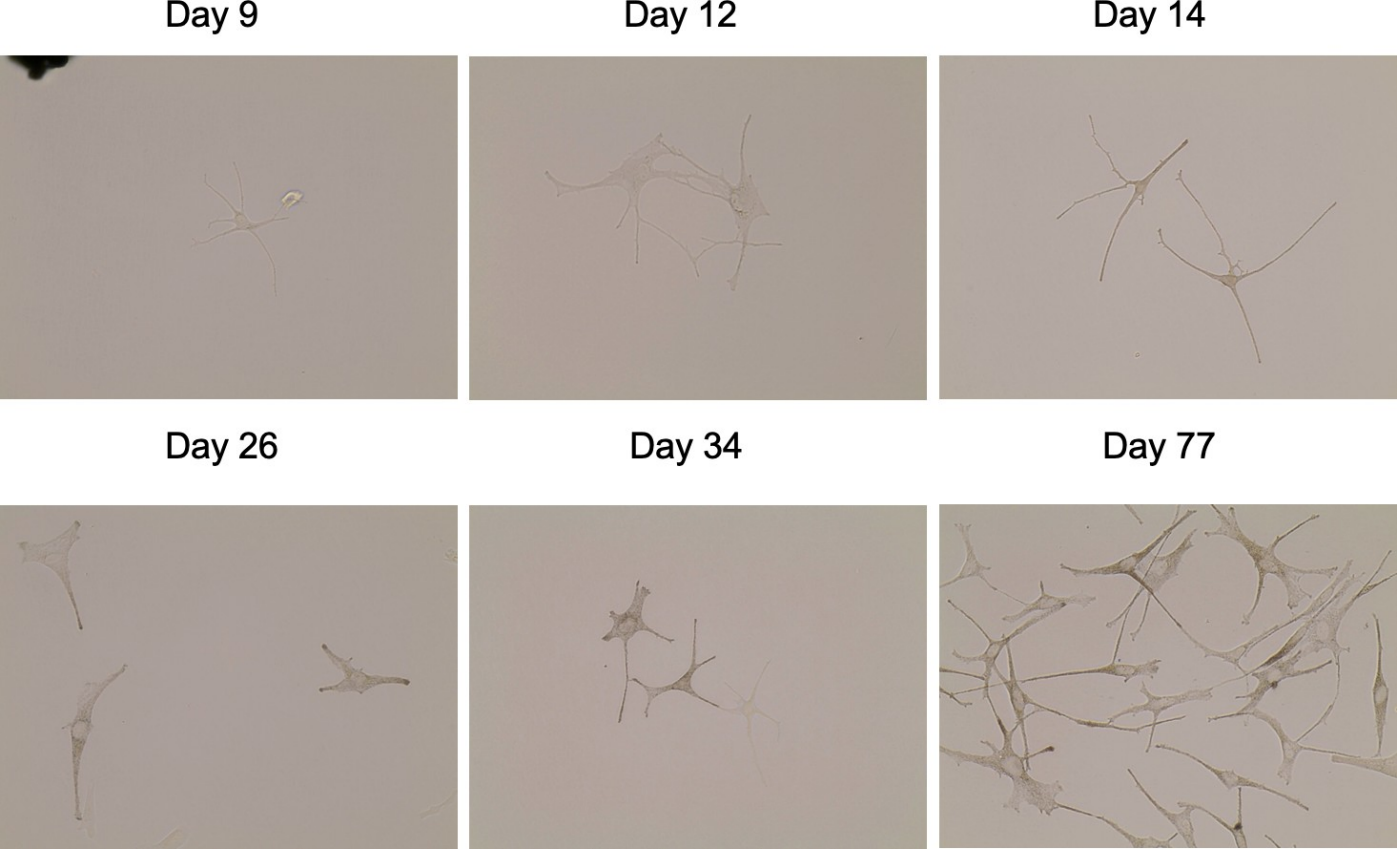
Melanocytes isolated from hypo-pigmented scar can be stimulated to produce melanin in media containing α-MSH. Punch biopsies of distinct regions of hyper- and hypo-pigmented scar were taken during a pre-planned surgical excision and were treated with dispase to isolate epidermal cells which were seeded in culture in media containing α-MSH. Images were taken under bright field microscopy at a fixed light intensity for 77 days Hypopigmented melanocytes over time are shown (B).

When cells were initially seeded into primary cell culture, hypopigmented cells did not contain a lot of melanin, and were easily viewable under phase contrast microscopy, but were very lightly pigmented such that they were undiscernible by bright field microscopy until imaging at day 9 (S15 and S16 Figs). In contrast, hyperpigmented melanocytes contained melanin and could be viewed under bright field microscopy. Over time, hypopigmented cells proliferated and re-pigmented, and melanin was identifiable by bright field microscopy in the cells at days 14, 34, and 77 in culture. Pigmentation in the once hypopigmented cells progressed to a level that was similar to the hyperpigmented cells, but never reached that of hyperpigmented levels. There was a steady increase in pigmentation over time (Fig 13).

#### 2.2.7 Hypopigmented melanocytes can be stimulated to produce melanin by NDP α-MSH through the up-regulation of TYR, TYRP1, and DCT

**Fig 14:**
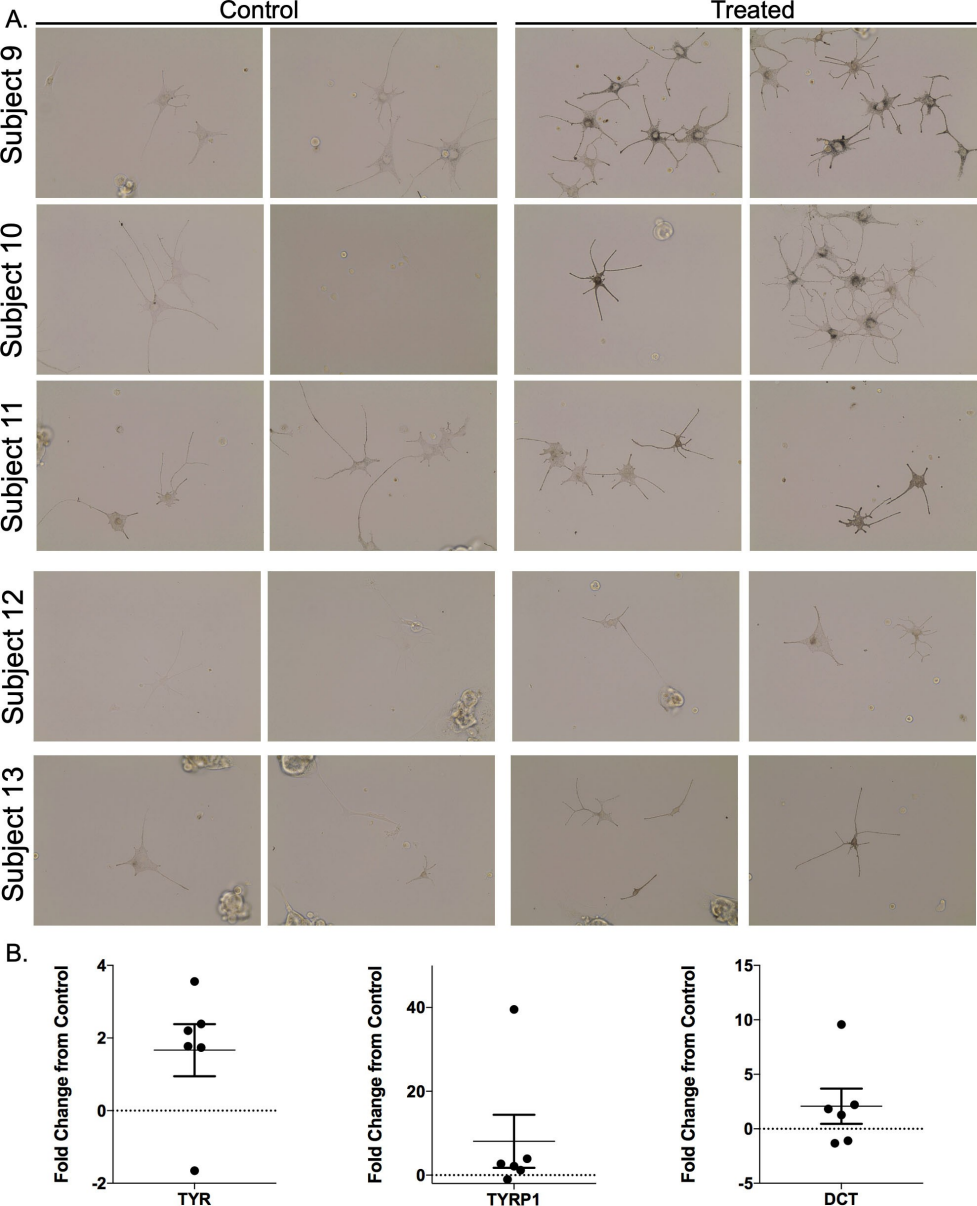
Melanocytes isolated from hypo-pigmented scar can be stimulated to produce melanin through the up-regulation of TYR, TYRP1, and DCT gene expression by exogenous treatment with NDP α-MSH. Punch biopsies of distinct regions of hypopigmented scar were taken and treated with dispase to isolate epidermal cells which were seeded in culture in media that did not contain α-MSH. After 3 days in culture, cells were treated with 10 μM NDP α-MSH for 72 hours. Images were taken under bright field microscopy at a fixed light intensity after 72 hours. RNA was isolated and qRT-PCR was performed for TYR, TYRP1, and DCT. GAPDH was used as a housekeeping control (mean ± SEM, n = 6) (B).

In each of the 6 cell lines derived from the different patients, when hypo-pigmented cells were treated with exogenous NDP α-MSH in CNT-40(s) media, there was an increase in pigmentation that was identifiable by bright field microscopy in the treated wells. The controls, which were treated with equal volumes of water, did not show this increase in melanin (Fig 14A). Treated cells had up-regulated levels of TYR, TYPR1, and DCT compared to controls (TYR = 1.67 ± 0.72; TYRP1 = 8.07 ± 6.33; DCT = 2.08 ± 1.62-fold change over control, n = 6) (Fig 14B). Each patient was normalized to their own control levels of gene expression without treatment. Average levels of fold change above a cut-off = 1.5 were accepted as gene up-regulation when analyzing data, however, a statistical comparison was not done due to the variable transcript expression among different patients which precluded using a single control. Gene expression up-regulation was variable among patients where some patient cells had up-regulation of all 3 genes (Subject #11, TYR = 2.39, TYRP1 = 2.69, DCT = 2.21). Some patient cells had up-regulation of two out of the three genes (Subject #10, TYR = 1.65, TYRP1 = 3.90, and DCT = 9.58). Some patient cells only showed up-regulation in one of the 3 genes (Subject #12, TYR = 3.56, TYPR = -1.03, DCT = 1.27).

## 3 Discussion

Dyspigmented HTS obtained from burn patients has many similarities to dyspigmented HTS obtained from a red Duroc pig animal model of scar following excisional wounding. The overall histological structure of the hyper- and hypo-pigmented scar was compared to the normal skin to show the hallmark characteristics of HTS in these tissue samples.

Another similarity between porcine and patient samples is the presence of melanocytes in regions of both hyper- and hypo-pigmentation in similar cell densities, as identified by *en face* staining; these numbers were slightly higher compared with those in the literature for normal human skin [13]. Hyperpigmented melanocytes have an “activated” phenotype with many dendrites present, while hypopigmented melanocytes have an “inactivated” phenotype with almost no dendrites which is consistent with a recently published paper by Dutta *et*. *al* [14]. Overall, the results would suggest that the use of the red Duroc pig animal model of dyspigmented HTS is useful moving forward, and may be most useful in the study of treatment mechanisms for this pathophysiology, which are currently poorly understood. It is possible that prevention strategies may need to be studied using a burn model of HTS so that the initiator is more similar to the patient condition; however, this study did not address this question specifically.

This study contained research subjects whose biopsies were stored and used in two different ways. These two groups were required because the amount of tissue biopsies that were collected was based on scar size. If the scar was >100 cm^2^, 2 tissue biopsies could be collected from each pigmentation phenotype. One biopsy was bisected and used for histology and molecular analysis. The other biopsy was processed with dispase to obtain an epidermal sheet. In the first group, all patients (n = 8) had the epidermal sheets fixed in MeOH for *en face* staining. In the second group, all epidermal sheets were used to isolated primary cells (n = 6).

Primary cell culture of melanocytes with dendritic morphology is another confirmation of the presence of these cells in hypopigmented scar. The cells of interest are confirmed to be melanocytes based on their morphology. It was noted that melanocyte morphology in hypopigmented regions of intact epidermal sheets by *en face* staining seemed to be inactive with no or few dendrites being present. When melanocytes synthesize melanin, it is transferred to keratinocytes that reside in the upper layers of the skin to protect against further DNA damage. Eumelanin is transferred along dendrites, which are formed specifically to facilitate this transfer [15–17]. Therefore, dendrite presence can act as a surrogate for melanocyte activity.

This activation status is subject to change, as was demonstrated when the cells were seeded into culture medium containing melanogenesis agonists such as α-MSH that stimulated dendritogenesis in hypopigmented melanocytes. This finding was the first observed positive data towards the hypothesis that these cells can be reprogrammed to produce melanin. It was of course possible that hypopigmented melanocytes are a cell type that no longer function and could never re-gain pigment or proliferate. On the contrary, it was hypothesized that hypopigmented melanocytes are functional. From the literature, it is clear that normal melanocytes that are stimulated with α-MSH upregulate transcription of MC1R to have receptors that can bind the ligand that was exogenously administered [18]. If hypopigmented melanocytes retain this ability to up-regulate MC1R, it may be the case that hypopigmented lesions may be re-pigmented through the use of pigmentation initiators [10].

The administration of α-MSH to the hypopigmented cells in the sample that was passively collected was not intentional. After it was observed that the cells gained dendrites after plating, the manufacturer of Cnt-40 media (Cellntec) was contacted, and it was disclosed that there are low levels of α-MSH (0.12 μM) in the basal media. This low level of α-MSH did not result in drastic changes in pigmentation during a short time-course. However, over a period of 2 months, cells re-gained pigmentation to similar levels as the hyperpigmented cells that were grown in a parallel time course. It is also possible that the media contained additional non-canonical agonists such as those discussed in the introduction. The logical criticism to this work is that there were no cells that were grown without α-MSH in the media. Therefore, additional tissue biopsies were collected, and these cells were grown in specialized media from the manufacturer that did not contain α-MSH. These cells were exogenously treated with 10 μM NDP α-MSH for a total of 72 hours. There was a marked increase in melanin in the treated cells, and no such increase in the control cells. This increase in melanin was confirmed to be through the upregulation of the 3 genes required for melanogenesis, TYR, TYRP1, and DCT. Variability in up-regulation of these genes indicates that in future work, multiple timepoints post-treatment should be investigated. In addition, it is possible that some hypopigmented cells only up-regulate certain genes and through an unknown mechanism cannot up-regulate all 3. This data is fodder for future work.

In an attempt to answer the above questions, pig melanocytes from regions of hypopigmentation were attempted to be isolated multiple times using a multitude of protocols. When these cells were grown in co-culture with keratinocytes, keratinocytes over-took the culture, and investigation into TYR, TYRP1, and DCT gene expression after treatment with NDP α-MSH did not show high enough cycle thresholds to evaluate these genes, suggesting that the melanocyte:keratinocyte ratio in this co-culture was too small. Analysis with brightfield microscopy likewise did not show changes in visual pigment between controls and treated cells. Melanocytes were attempted to be isolated from the keratinocytes by differential trypsinization techniques as described above in the patient samples, however it was not as effective in eliminating keratinocytes. Pig keratinocytes also had a much higher tolerance to treatment with calcium, yielding difficulties with differentiation of keratinocytes out of melanocyte cultures. Lastly, cell sorting by magnetic or fluorescence-activated cell sorting was going to be attempted, however, a suitable marker for all melanocytes (regardless of pigmentation status) could not be found. MART/MELAN-A was not suitable because it is transcriptionally activated by MiTF, and would not have been activated in hypopigmented cells as was previously shown by at the gene level in prior work [19]. S100β, the marker used for *en face* staining [20,21], does not show differences in protein expression based on the pigmentation phenotype, and hence, would have been a suitable marker, however, it is not a membrane protein, and hence, not suitable for sorting live cells for culture [22]. On-going work is aimed at further optimizing strategies for culturing hypopigmented melanocytes from pig scars to provide a large cell pool with which to study treatments at a more in-depth level.

These data further confirm the presence of melanocytes in hypopigmented regions of HTS in pig and patient samples. It also confirms that hypopigmented melanocytes can be stimulated with NDP α-MSH to regain pigmentation *in vitro*. The *in vitro* environment is different from the *in vivo* HTS environment, and does not entirely recapitulate the multitude of cell types and non-cellular components that may contribute to dyschromia. Therefore, the next logical step in future work will be to test the treatment of hypopigmented scar cells with NDP α-MSH in an *in vivo* system. This data supports the notion that tissue-sparing techniques where hypopigmented scars can be treated topically with NDP α-MSH may be developed in the future to treat this symptom of scar that continues to be challenging for patients both aesthetically and psychosocially.

## 4 Materials and methods

### 4.1 Duroc pig model

All animal work was approved by the MedStar Health Research Institute’s Institutional Animal Care and Use Committee. Dyschromic hypertrophic scars (n = 3 per pig) were created on the flanks of Duroc pigs by full thickness excisional wounding by dermatome as previously described [9]. Sample sizes were reflective of those needed to acquire pilot data, and no power calculation was performed. During all animal procedures, heart rate and oxygen saturation were monitored during induction. During the procedures, heart rate, blood pressure, respiratory rate, end tidal CO_2_, oxygen saturation, and temperature were monitored and adjusted to ensure appropriate planes of anesthesia. On the wound creation days, warmed intravenous maintenance fluids were administered. Areas of distinct hyper-, hypo-, and normal pigmentation were biopsied at Day 56 post-excision. At the final timepoint, animals were given an intravenous dose of 4.2M potassium chloride solution at 2 mmol/kg for euthanasia and death was confirmed with flatline vital signs. There were no unanticipated adverse events during these procedures.

### 4.2 Patient enrollment and sample collection

#### 4.2.1 Passive tissue collection

During a scar excision and grafting procedure 15 months post-burn, HTS was collected under an approved IRB protocol (study #2012–338) for discarded skin. The scar was processed in the laboratory where 3 mm punch biopsies were taken of hyper- and hypo-pigmented areas, as well as of normal skin. These biopsies were formalin-fixed and paraffin embedded (FFPE) for histological assays. Larger pieces of hyper- or hypo-pigmented scar areas were sterilized and processed to obtain epidermal sheets for tissue culture as described below.

*Active tissue collection*. All patients were consented and enrolled under a protocol approved by the MedStar Health Research Institute’s Institutional Review Board (IRB) under study #00000430. Inclusion criteria included males and females greater than 18 years of age with cutaneous trauma resulting in dyschromic scar. Exclusion criteria included dyschromic scar solely to the face, genitalia, or hands, known allergy to lidocaine, pregnancy, and/or prisoner status. Digital images of patient scars were captured and patients gave their consent for these images to be published. Non-invasive skin probes were used to measure melanin using a skin color catch (SCC) probe (Delfin Technologies, Stamford, CT). 3 mm punch biopsies of distinct regions of hyper- and hypo-pigmented scar and normally pigmented skin were collected and processed as described below. A subset of patients had epidermal sheets fixed in methanol for *en face* staining (n = 9), while a subset of patients had epidermal sheets processed for cell culture (n = 6).

#### 4.2.2 Histology

FFPE biopsies were sectioned at 6 μm, and stained with H&E or Fontana-Masson as previously described [4].

#### 4.2.3 Generation of epidermal sheets

3 mm punch biopsies were sterilized through successive washes with 100% ethanol (2X), water, and PBS (2X). They were then submerged in 1X dispase solution (CELLnTEC, Switzerland) with 10 μg/mL gentamycin and 0.25 μg/mL amphotericin B and were incubated overnight at 4°C. The following day, the epidermis was peeled from the dermis. It was fixed in ice-cold methanol for 10 minutes for *en face* staining or it was used for primary cell culture.

#### 4.2.4 En face staining

Hyper- and hypo-pigmented epidermal sheets were stained with a melanocyte marker, S100β by an *en face* staining technique adapted from a previously described method [23]. The protocol used a primary S100β antibody diluted in 3% milk (1:50; ab11178) (Abcam, Cambridge, MA) and a secondary antibody (anti-mouse-CY3 at 1:100; Abcam) with subsequent DAPI application [24]. During the course of the study, the production of one S100β antibody was discontinued by the company, and the antibody was replaced with a new one (1:100; ab52642), and stains were completed with anti-rabbit-CY3 secondary antibodies. For the pig model, n = 9 scars from n = 3 pigs were sampled to generate hyper- and hypo-pigmented epidermal sheets. Within each sheet, 5 high-powered fields (HPF) were imaged, and cells were counted at 40X magnification. Within each HPF, dendrites were counted in a representative cell in each picture to obtain an average count per HPF. For the patient samples, n = 8 scars from n = 8 patients were sampled to generate hyper- and hypo-pigmented epidermal sheets. Cells and dendrites were counted as described above.

#### 4.2.5 Primary cell culture in media containing α-MSH

Cells were isolated as previously described [4]. Briefly, epidermal sheets were disaggregated by pipetting to release single cells. The suspension was pelleted and reconstituted in CnT-40 complete media that contained 0.2 μg/mL (0.120 μM) α-MSH. After the cells were allowed to attach overnight, melanocytes were selected from the keratinocyte-melanocyte co-culture by differential trypsinization of melanocytes with 0.00125% trypsin diluted in EDTA for 2 minutes. Cells were then seeded onto a 10-fold reduced surface area with 1.4 mM calcium chloride to differentiate any keratinocytes and remove them from the culture. Media was then changed every other day and cells were imaged under bright field microscopy with consistent lighting throughout a 77-day time course. Hypopigmented melanocytes proliferated more slowly compared to hyperpigmented melanocytes. To achieve confluence, these cells had to be grown for extended periods of time. Fibroblast cells contaminated the culture at day 22, and fibroblasts were removed from the cultures using magnetic activated cell sorting with anti-fibroblast beads (Milltenyi Biotec, Bergisch Gladbach, Germany, 130-050-601). This process was carried out according to the manufacturer’s protocol for LS columns.

*Primary cell culture in media that did not contain α-MSH and cell treatments*. Cells were isolated as described above [4]. Briefly, epidermal sheets were disaggregated by pipetting to release single cells. The suspension was pelleted and reconstituted in specialized CnT-40 media (CNT-40(s)) that was ordered from the manufacturer and did not contain α-MSH. The cells resulting from each 3 mm punch biopsy were seeded into 2 wells of a twelve-well plate (Corning, Tewksbury, MA). Non adherent cells were rinsed after 48 hours.

Cells were grown in fresh CNT-40(s) media for 72 hours. Media was then aspirated, and one of the hypopigmented wells was treated with 10 μM NDP α-MSH (Tocris Bioscience, Bristol, UK). The untreated well had an equivalent volume of water added. Fresh media containing treatment or control was replaced at 24 and 48 hours after the initiation of treatment. At 72 hours, cells were imaged under brightfield microscopy, and cells were lysed with Trizol Reagent (Thermo Fisher Scientific, Halethorpe, MD).

#### 4.2.6 RNA Isolation and qRT-PCR

RNA was isolated from cells using a phenol-chloroform extraction and purification method as previously described [4]. qRT-PCR was performed using primers for TYR (NM_000372.4), TYRP1 (NM_000550.2), and DCT (NM_001922.3) (Qiagen, Valencia, CA). GAPDH (NM_002046.7) was used as a housekeeping gene (Forward primer: 5’-CAA TGA CCC CTT CAT TGA CCT C -3’, reverse primer: 5’-AGC-ATC-GCC-CCA-CTT-GAT-T-3’). Reactions were cycled as previously described [25]. The ΔΔC_t_ method was used to analyze the data with the untreated hypopigmented cells as the control. Up-regulation of genes was defined as an average fold change >1.5 from control cells.

#### 4.2.7 Statistical analysis

When comparing melanin indices from pig or patient scars between hyper-, hypo-, and normally pigmented areas, a one-way ANOVA was used with Tukey’s correction for multiple comparisons. When comparing melanocyte number and melanocyte dendricity from pig or patient scars between hyper- and hypo-pigmented areas, an un-paired Student’s t-test was used. When analyzing the qRT-PCR data, a fold change cut-off>1.5 was set, but a statistical comparison was not performed because each patient was normalized to their own control levels of gene expression without treatment. Significance was set at p<0.05 for all tests.

#### 4.2.8 Conclusions

In dyschromic HTS from Duroc pigs and patients, regions of hypopigmented scar contain melanocytes in equal quantity to hyperpigmented areas. In patient samples, hypopigmented regions of scar contain “inactive” melanocytes that can be stimulated to make melanin by α-MSH *in vitro*.

## Supporting information

S1 Fig Regions of hyper- and hypo-pigmentation share many of the hallmark characteristics of HTS compared to normal skin. Hyper- and hypo-pigmented scar and normal skin FFPE biopsies were stained with H&E. Scale bar = 500 μm at 1.25X (A), 100 μm at 5X (B), 50 μm at 10X (C), and 20 μm at 40X (D). Bracket indicates thickness. White circles indicate blood vessels. Black circles indicate collagen disorganization. Arrows indicate presence of rete ridges.
(TIF)

S2 Fig Regions of hyper- and hypo-pigmentation contain melanocytes in equal numbers. Epidermal sheets from regions of hyper- or hypo-pigmentation were stained for melanocyte marker, S100β by *en face* staining. S100β (red), DAPI (blue). Scale Bar = 50 μm at 10X (left) or 10 μm at 40X (right) (A). Images are from Subject #2 from Table 2. (Scale bar = 50 μm for 10X, top and 20 μm for 40X, bottom).
(TIF)

S3 Fig Regions of hyper- and hypo-pigmentation contain melanocytes in equal numbers. Epidermal sheets from regions of hyper- or hypo-pigmentation were stained for melanocyte marker, S100β by *en face* staining. S100β (red), DAPI (blue). Scale Bar = 50 μm at 10X (left) or 10 μm at 40X (right) (A). Images are from Subject #3 from Table 2. (Scale bar = 50 μm for 10X, top and 20 μm for 40X, bottom).
(TIF)

S4 Fig Regions of hyper- and hypo-pigmentation contain melanocytes in equal numbers. Epidermal sheets from regions of hyper- or hypo-pigmentation were stained for melanocyte marker, S100β by *en face* staining. S100β (red), DAPI (blue). Scale Bar = 50 μm at 10X (left) or 10 μm at 40X (right) (A). Images are from Subject #4 from Table 2. (Scale bar = 50 μm for 10X, top and 20 μm for 40X, bottom).
(TIF)

S5 Fig Regions of hyper- and hypo-pigmentation contain melanocytes in equal numbers. Epidermal sheets from regions of hyper- or hypo-pigmentation were stained for melanocyte marker, S100β by *en face* staining. S100β (red), DAPI (blue). Scale Bar = 50 μm at 10X (left) or 10 μm at 40X (right) (A). Images are from Subject #5 from Table 2. (Scale bar = 50 μm for 10X, top and 20 μm for 40X, bottom).
(TIF)

S6 Fig Regions of hyper- and hypo-pigmentation contain melanocytes in equal numbers. Epidermal sheets from regions of hyper- or hypo-pigmentation were stained for melanocyte marker, S100β by *en face* staining. S100β (red), DAPI (blue). Scale Bar = 50 μm at 10X (left) or 10 μm at 40X (right) (A). Images are from Subject #6 Table 2. (Scale bar = 50 μm for 10X, top and 20 μm for 40X, bottom).
(TIF)

S7 Fig Regions of hyper- and hypo-pigmentation contain melanocytes in equal numbers. Epidermal sheets from regions of hyper- or hypo-pigmentation were stained for melanocyte marker, S100β by *en face* staining. S100β (red), DAPI (blue). Scale Bar = 50 μm at 10X (left) or 10 μm at 40X (right) (A). Images are from Subject #7 from Table 2. (Scale bar = 50 μm for 10X, top and 20 μm for 40X, bottom).
(TIF)

S8 Fig Regions of hyper- and hypo-pigmentation contain melanocytes in equal numbers. Epidermal sheets from regions of hyper- or hypo-pigmentation were stained for melanocyte marker, S100β by *en face* staining. S100β (red), DAPI (blue). Scale Bar = 50 μm at 10X (left) or 10 μm at 40X (right) (A). Images are from Subject #8 from Table 2. (Scale bar = 50 μm for 10X, top and 20 μm for 40X, bottom).
(TIF)

S9 Fig Staining reveals structural architecture and melanin deposition in hyper- and hypo-pigmented patient HTS compared to normal skin. Melanocytes were cultured from biopsies regardless of pigmentation phenotype. Punch biopsies of distinct regions of hyper- (I), hypo- (II), and normally-pigmented (III) scar and skin were taken and were FFPE and H&E stained. Images are from Subject #10 in Table 2. (Scale bar = 100 μm for 10X, and 20 μm for 40X).
(TIF)

S10 Fig Staining reveals structural architecture and melanin deposition in hyper- and hypo-pigmented patient HTS compared to normal skin. Melanocytes were cultured from biopsies regardless of pigmentation phenotype. Punch biopsies of distinct regions of hyper- (I), hypo- (II), and normally-pigmented (III) scar and skin were taken and were FFPE and Fontana-Masson stained (B). Images are from Subject #10 in Table 2. (Scale bar = 100 μm for 10X, and 20 μm for 40X).
(TIF)

S11 Fig Staining reveals structural architecture and melanin deposition in hyper- and hypo-pigmented patient HTS compared to normal skin. Melanocytes were cultured from biopsies regardless of pigmentation phenotype. Punch biopsies of distinct regions of hyper- (I), hypo- (II), and normally-pigmented (III) scar and skin were taken and treated with dispase to isolate epidermal cells which were seeded in culture. Images were taken under bright field microscopy at a fixed light intensity after 3 days in culture. Images are from Subject #10 in Table 2. (Scale bar = 100 μm for 10X, and 20 μm for 40X).
(TIF)

S12 Fig Staining reveals structural architecture and melanin deposition in hyper- and hypo-pigmented patient HTS compared to normal skin. Melanocytes were cultured from biopsies regardless of pigmentation phenotype. Punch biopsies of distinct regions of hyper- (I), hypo- (II), and normally-pigmented (III) scar and skin were taken and were FFPE and H&E stained. Images are from Subject #11 in Table 2. (Scale bar = 100 μm for 10X, and 20 μm for 40X).
(TIF)

S13 Fig Staining reveals structural architecture and melanin deposition in hyper- and hypo-pigmented patient HTS compared to normal skin. Melanocytes were cultured from biopsies regardless of pigmentation phenotype. Punch biopsies of distinct regions of hyper- (I), hypo- (II), and normally-pigmented (III) scar and skin were taken and were FFPE and Fontana-Masson stained (B). Images are from Subject #11 in Table 2. (Scale bar = 100 μm for 10X, and 20 μm for 40X).
(TIF)

S14 Fig Staining reveals structural architecture and melanin deposition in hyper- and hypo-pigmented patient HTS compared to normal skin. Melanocytes were cultured from biopsies regardless of pigmentation phenotype. Punch biopsies of distinct regions of hyper- (I), hypo- (II), and normally-pigmented (III) scar and skin were taken and treated with dispase to isolate epidermal cells which were seeded in culture. Images were taken under bright field microscopy at a fixed light intensity after 3 days in culture. Images are from Subject #11 in Table 2. (Scale bar = 100 μm for 10X, and 20 μm for 40X).
(TIF)

S15 Fig Melanocytes isolated from hypo-pigmented scar can be stimulated to produce melanin in media containing α-MSH. Punch biopsies of distinct regions of hyper- and hypo-pigmented scar were taken during a pre-planned surgical excision and were treated with dispase to isolate epidermal cells which were seeded in culture in media containing α-MSH. Images were taken under bright field microscopy at a fixed light intensity at Days 9 and 14.
(TIF)

S16 Fig Melanocytes isolated from hypo-pigmented scar can be stimulated to produce melanin in media containing α-MSH. Punch biopsies of distinct regions of hyper- and hypo-pigmented scar were taken during a pre-planned surgical excision and were treated with dispase to isolate epidermal cells which were seeded in culture in media containing α-MSH. Images were taken under bright field microscopy at a fixed light intensity at Days 34 and 77.
(TIF)
