# Supplementary figures and images for: Hypopigmented burn hypertrophic scar contains melanocytes that can be signaled to re-pigment by synthetic alpha-melanocyte stimulating hormone in vitro
Source: PLoS One. 2021 Mar 25;16(3):e0248985. doi: 10.1371/journal.pone.0248985 (PMC7993611; doi:10.1371/journal.pone.0248985)

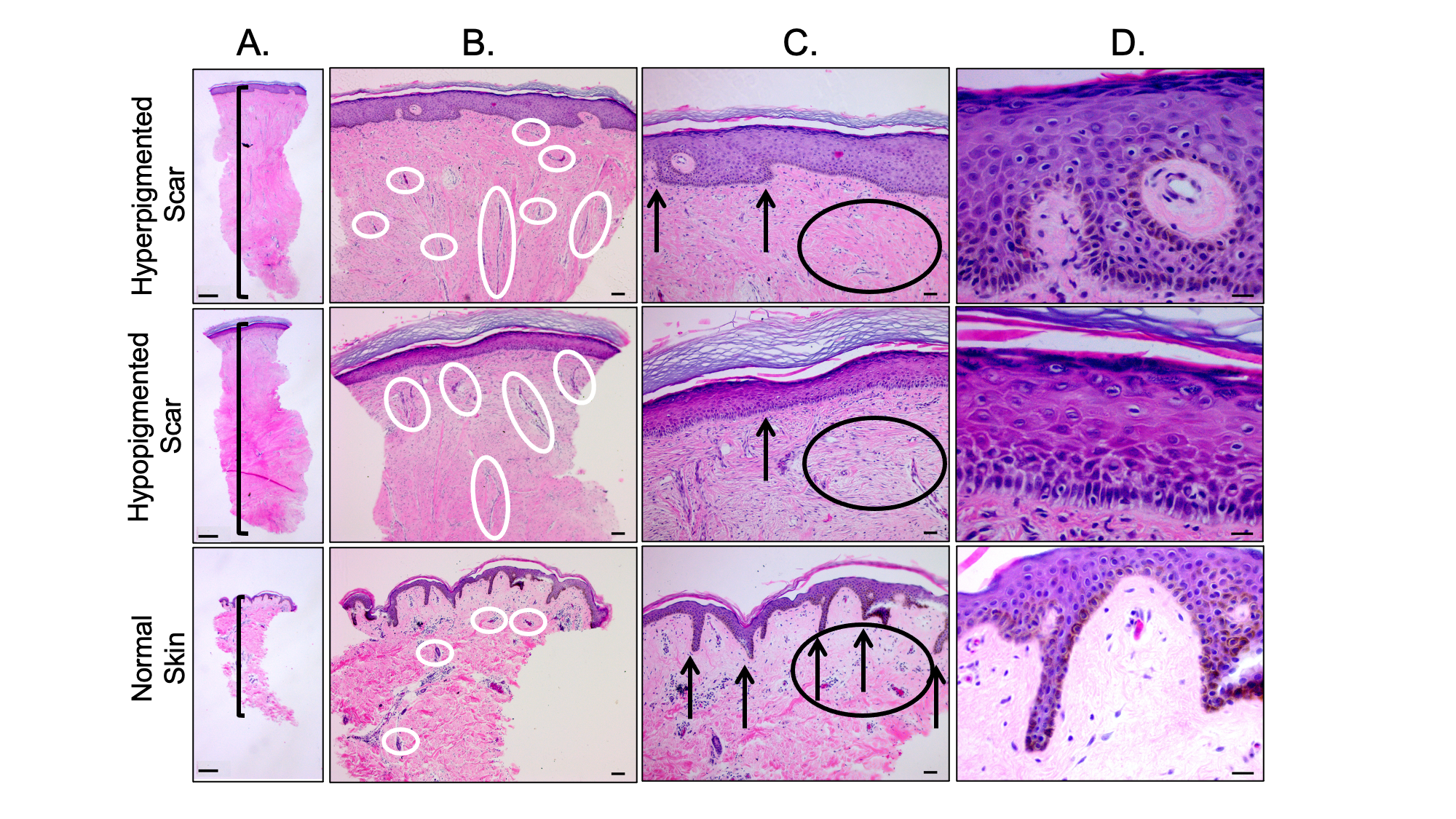

Supplement: S1 Fig — Hyper- and hypo-pigmented scar and normal skin FFPE biopsies were stained with H&E. Scale bar = 500 μm at 1.25X (A), 100 μm at 5X (B), 50 μm at 10X (C), and 20 μm at 40X (D). Bracket indicates thickness. White circles indicate blood vessels. Black circles indicate collagen disorganization. Arrows indicate presence of rete ridges. (TIF) [file pone.0248985.s001.tif]

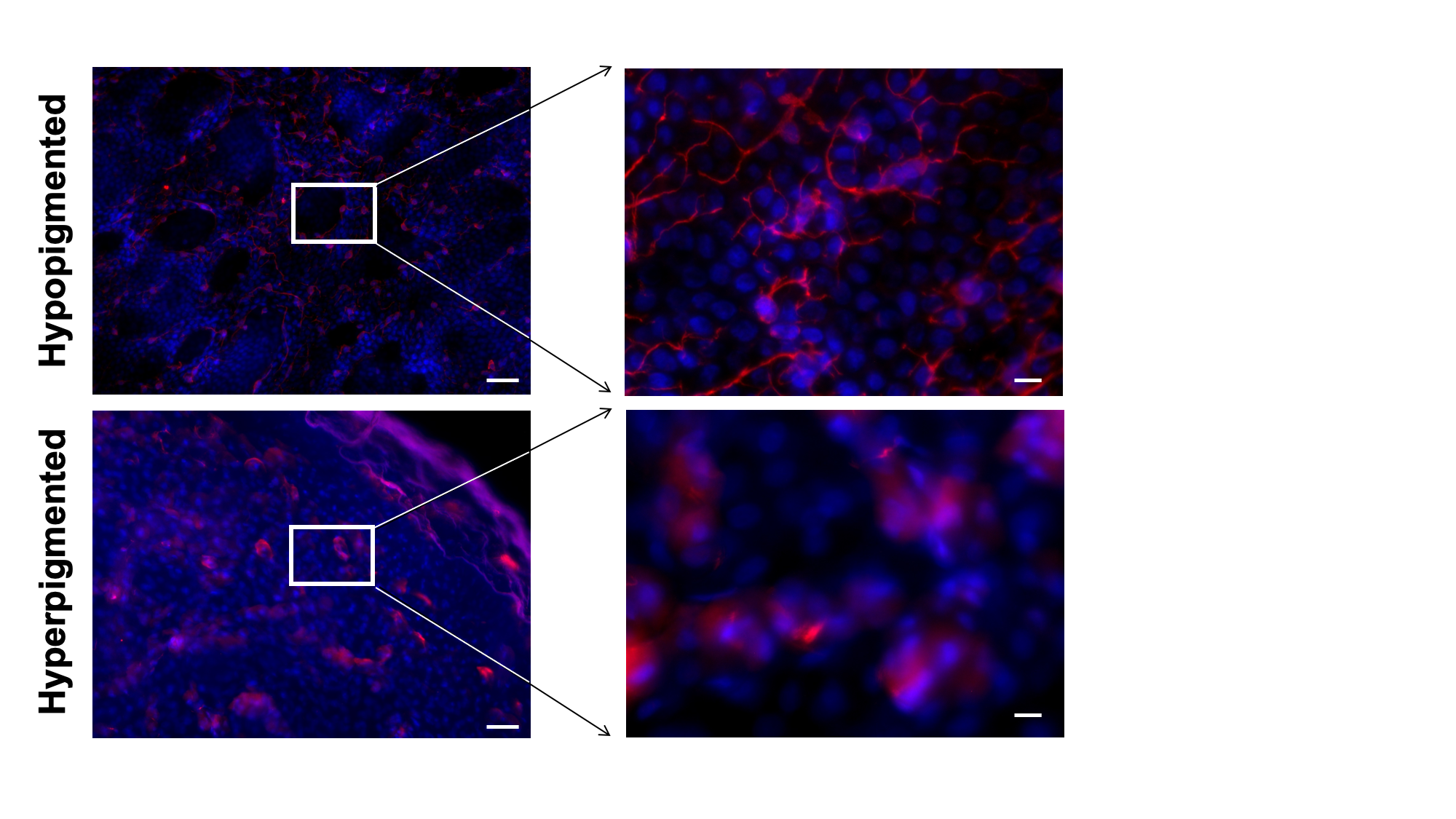

Supplement: S2 Fig — Epidermal sheets from regions of hyper- or hypo-pigmentation were stained for melanocyte marker, S100β by en face staining. S100β (red), DAPI (blue). Scale Bar = 50 μm at 10X (left) or 10 μm at 40X (right) (A). Images are from Subject #2 from Table 2. (Scale bar = 50 μm for 10X, top and 20 μm for 40X, bottom). (TIF) [file pone.0248985.s002.tif]

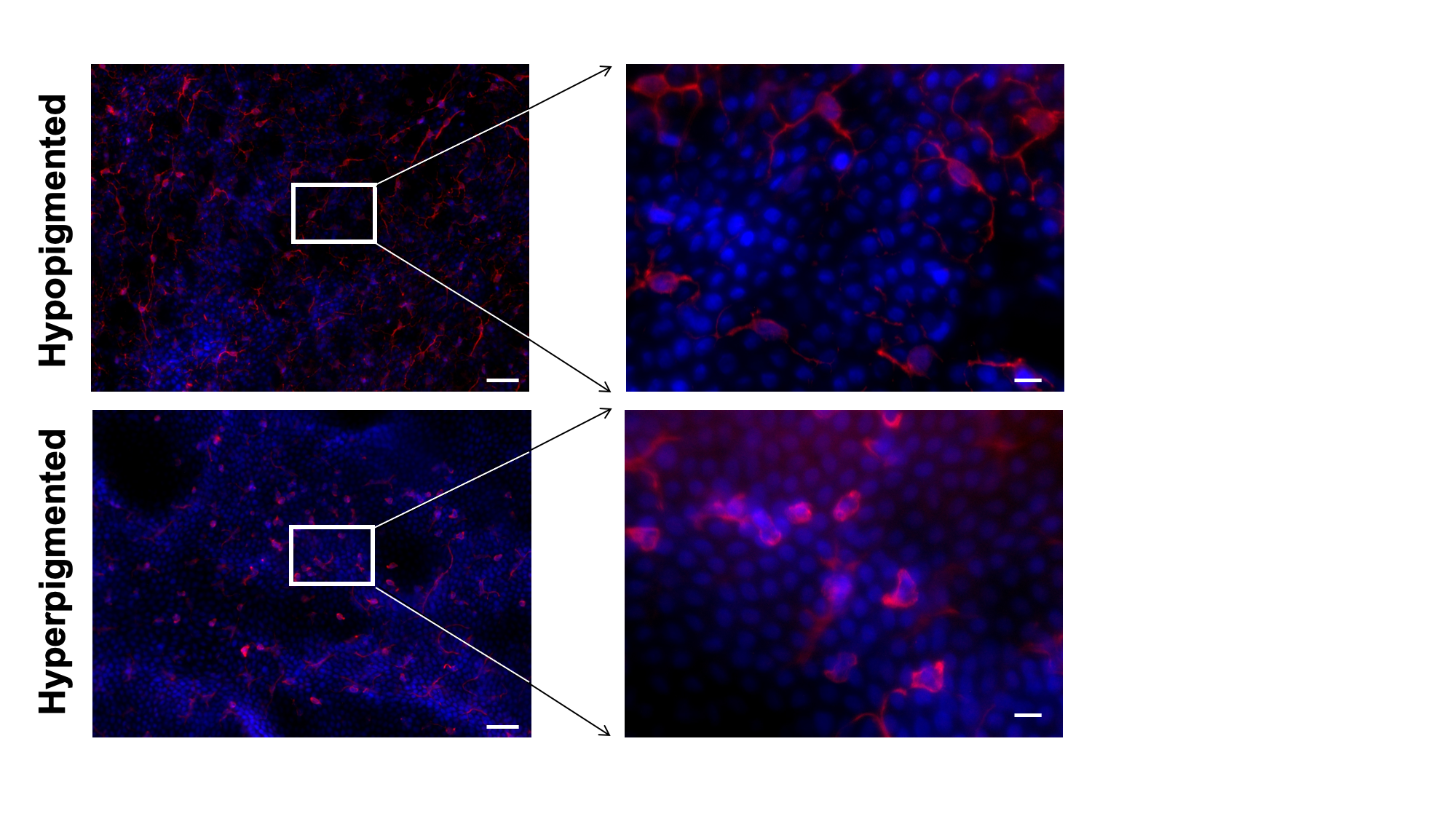

Supplement: S3 Fig — Epidermal sheets from regions of hyper- or hypo-pigmentation were stained for melanocyte marker, S100β by en face staining. S100β (red), DAPI (blue). Scale Bar = 50 μm at 10X (left) or 10 μm at 40X (right) (A). Images are from Subject #3 from Table 2. (Scale bar = 50 μm for 10X, top and 20 μm for 40X, bottom). (TIF) [file pone.0248985.s003.tif]

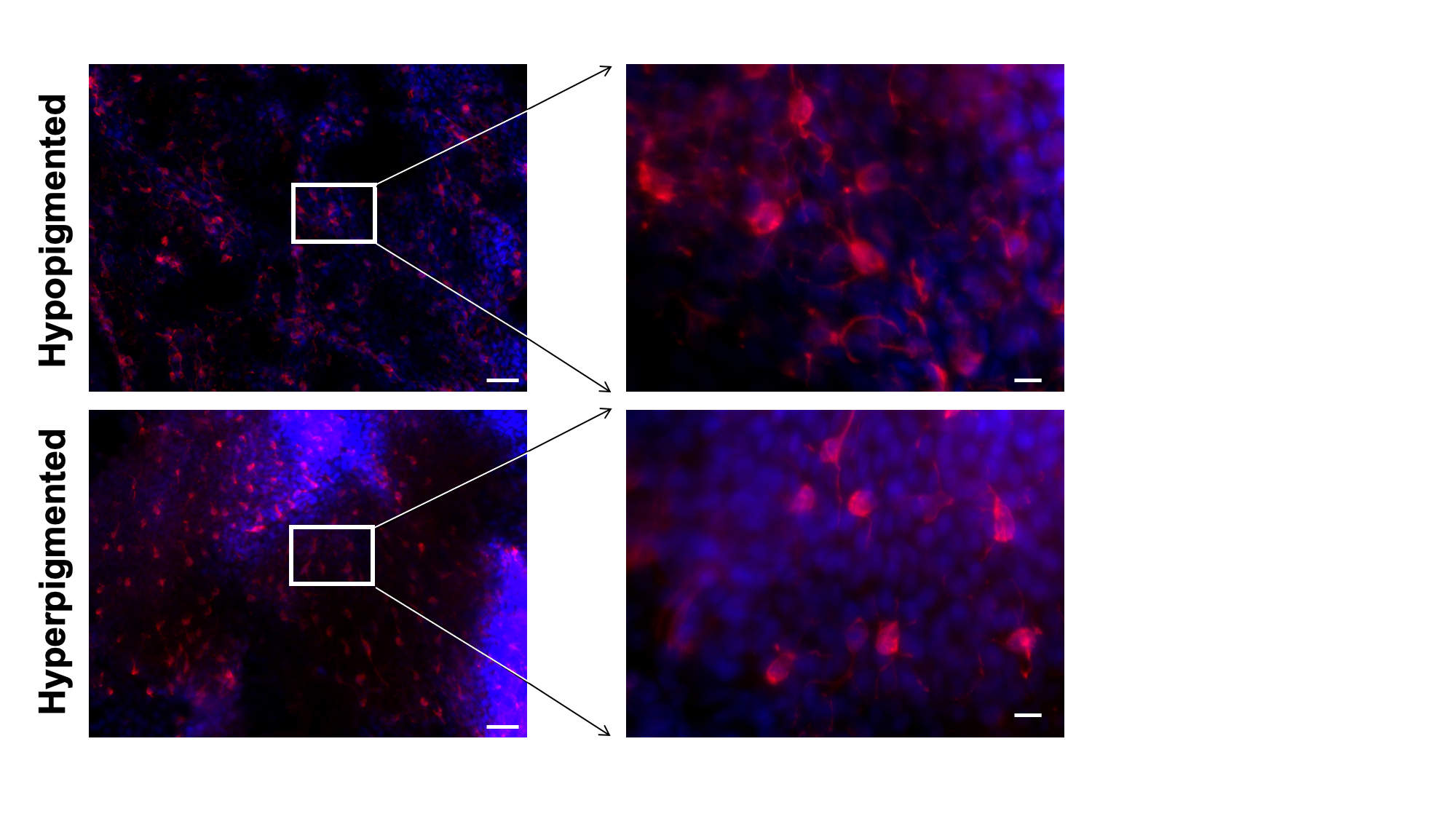

Supplement: S4 Fig — Epidermal sheets from regions of hyper- or hypo-pigmentation were stained for melanocyte marker, S100β by en face staining. S100β (red), DAPI (blue). Scale Bar = 50 μm at 10X (left) or 10 μm at 40X (right) (A). Images are from Subject #4 from Table 2. (Scale bar = 50 μm for 10X, top and 20 μm for 40X, bottom). (TIF) [file pone.0248985.s004.tif]

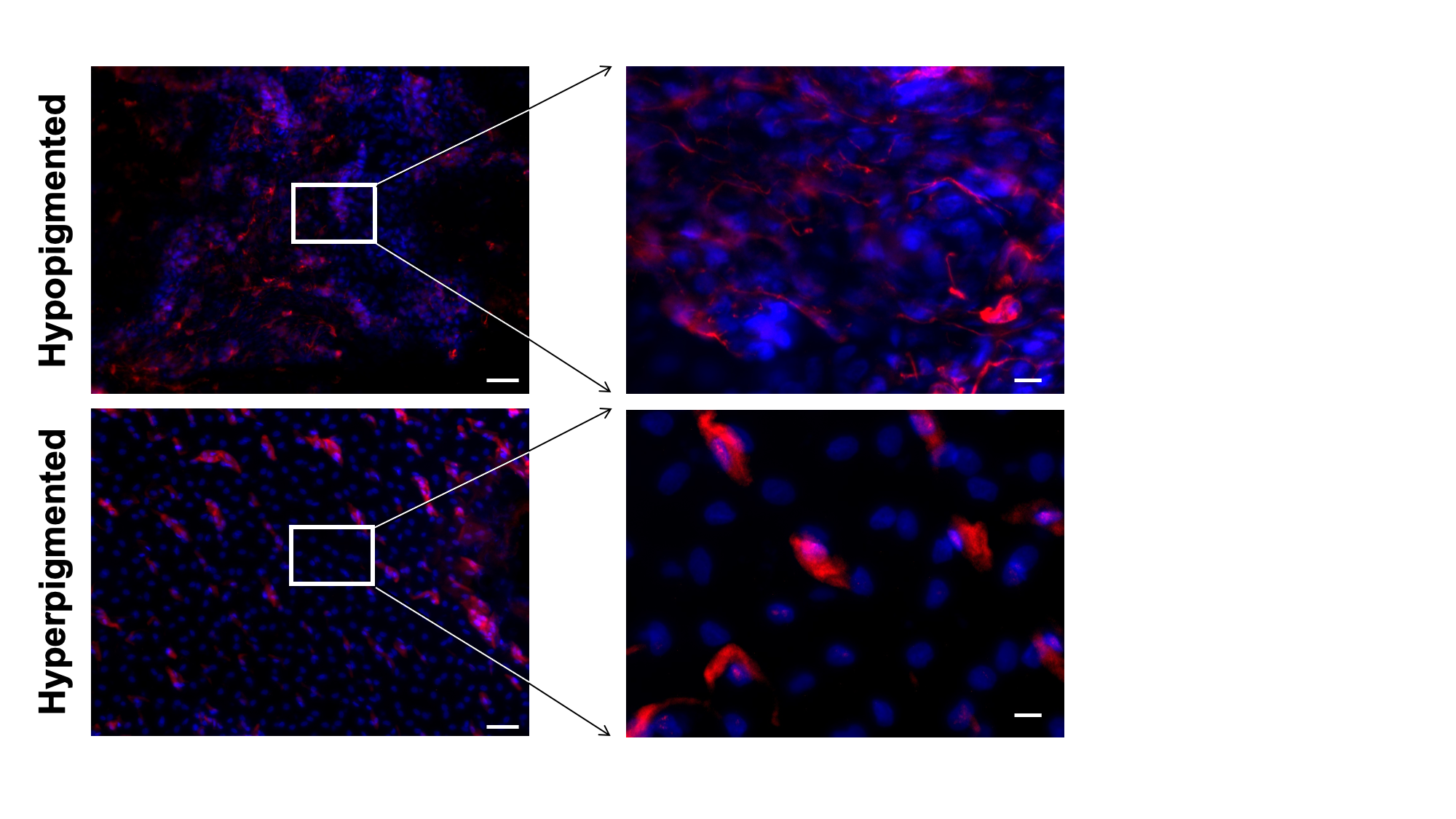

Supplement: S5 Fig — Epidermal sheets from regions of hyper- or hypo-pigmentation were stained for melanocyte marker, S100β by en face staining. S100β (red), DAPI (blue). Scale Bar = 50 μm at 10X (left) or 10 μm at 40X (right) (A). Images are from Subject #5 from Table 2. (Scale bar = 50 μm for 10X, top and 20 μm for 40X, bottom). (TIF) [file pone.0248985.s005.tif]

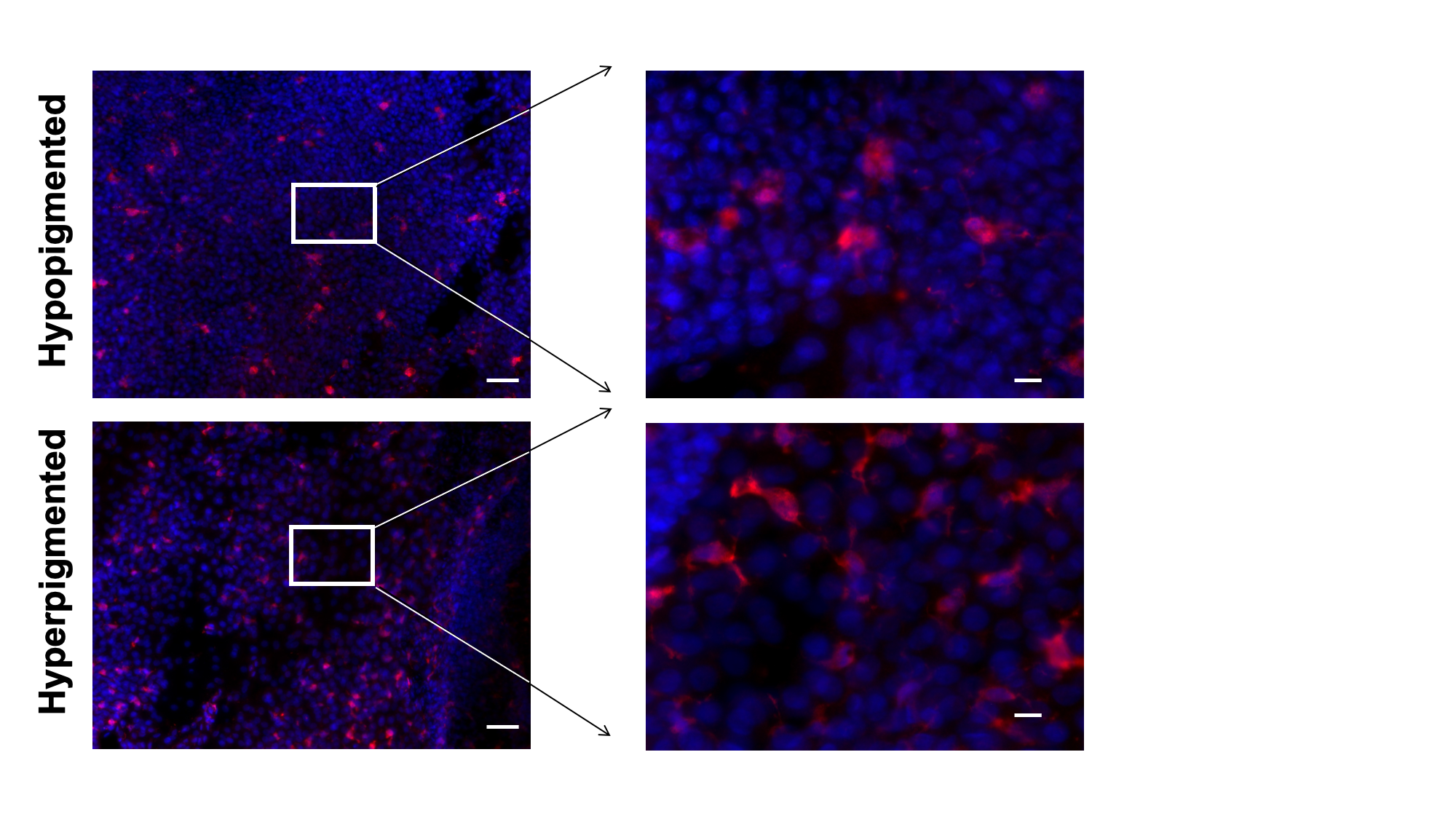

Supplement: S6 Fig — Epidermal sheets from regions of hyper- or hypo-pigmentation were stained for melanocyte marker, S100β by en face staining. S100β (red), DAPI (blue). Scale Bar = 50 μm at 10X (left) or 10 μm at 40X (right) (A). Images are from Subject #6 Table 2. (Scale bar = 50 μm for 10X, top and 20 μm for 40X, bottom). (TIF) [file pone.0248985.s006.tif]

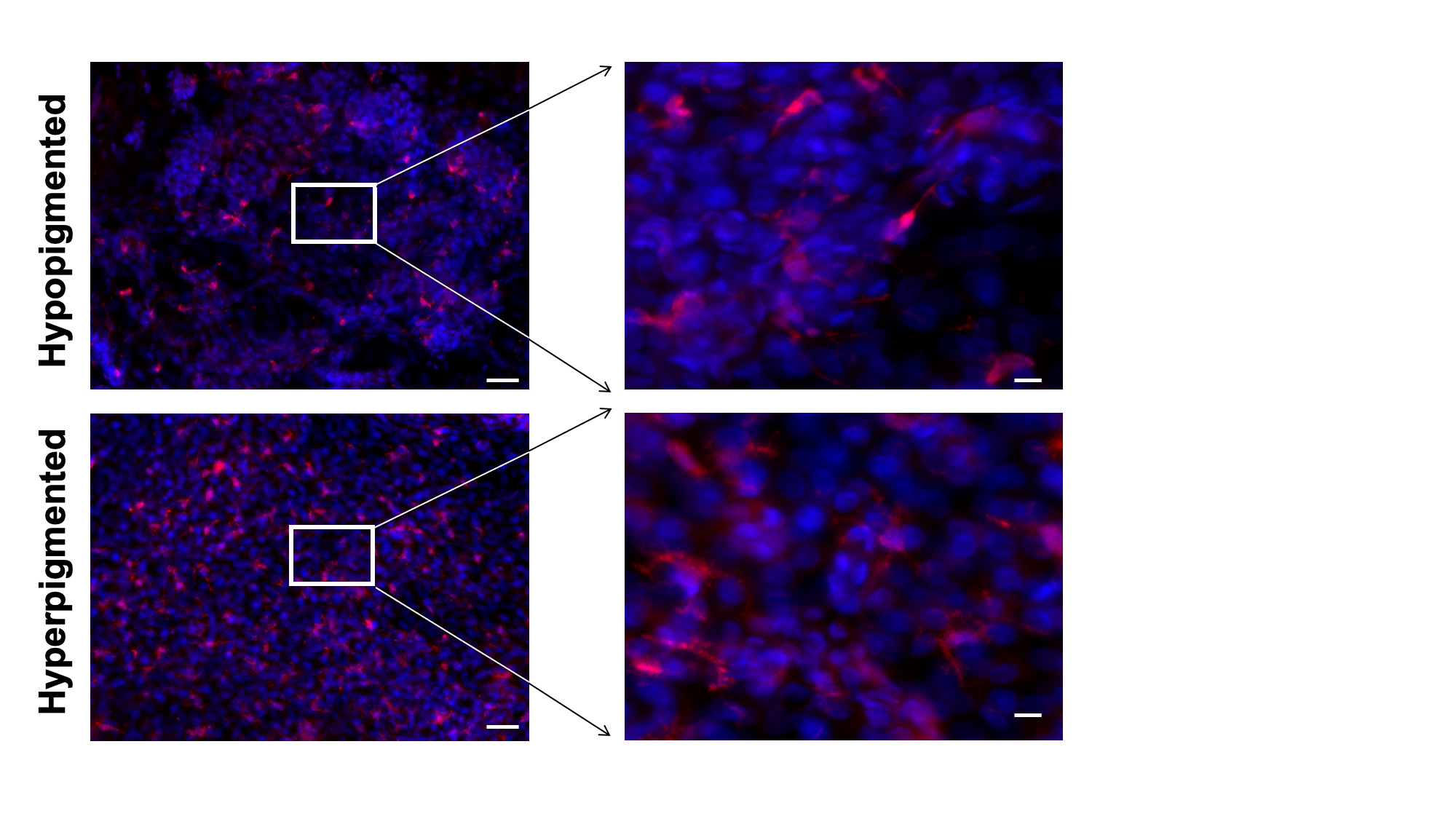

Supplement: S7 Fig — Epidermal sheets from regions of hyper- or hypo-pigmentation were stained for melanocyte marker, S100β by en face staining. S100β (red), DAPI (blue). Scale Bar = 50 μm at 10X (left) or 10 μm at 40X (right) (A). Images are from Subject #7 from Table 2. (Scale bar = 50 μm for 10X, top and 20 μm for 40X, bottom). (TIF) [file pone.0248985.s007.tif]

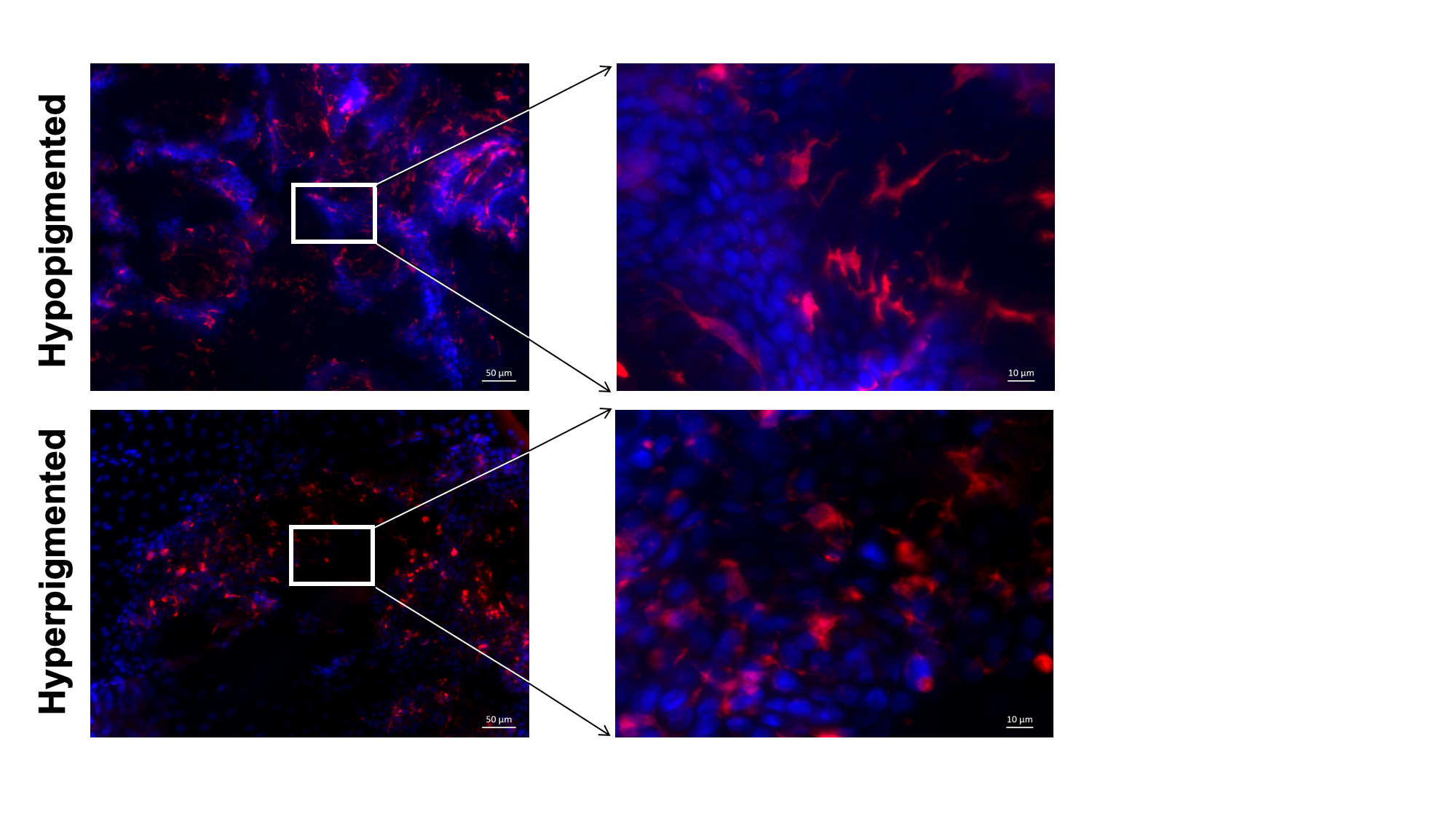

Supplement: S8 Fig — Epidermal sheets from regions of hyper- or hypo-pigmentation were stained for melanocyte marker, S100β by en face staining. S100β (red), DAPI (blue). Scale Bar = 50 μm at 10X (left) or 10 μm at 40X (right) (A). Images are from Subject #8 from Table 2. (Scale bar = 50 μm for 10X, top and 20 μm for 40X, bottom). (TIF) [file pone.0248985.s008.tif]

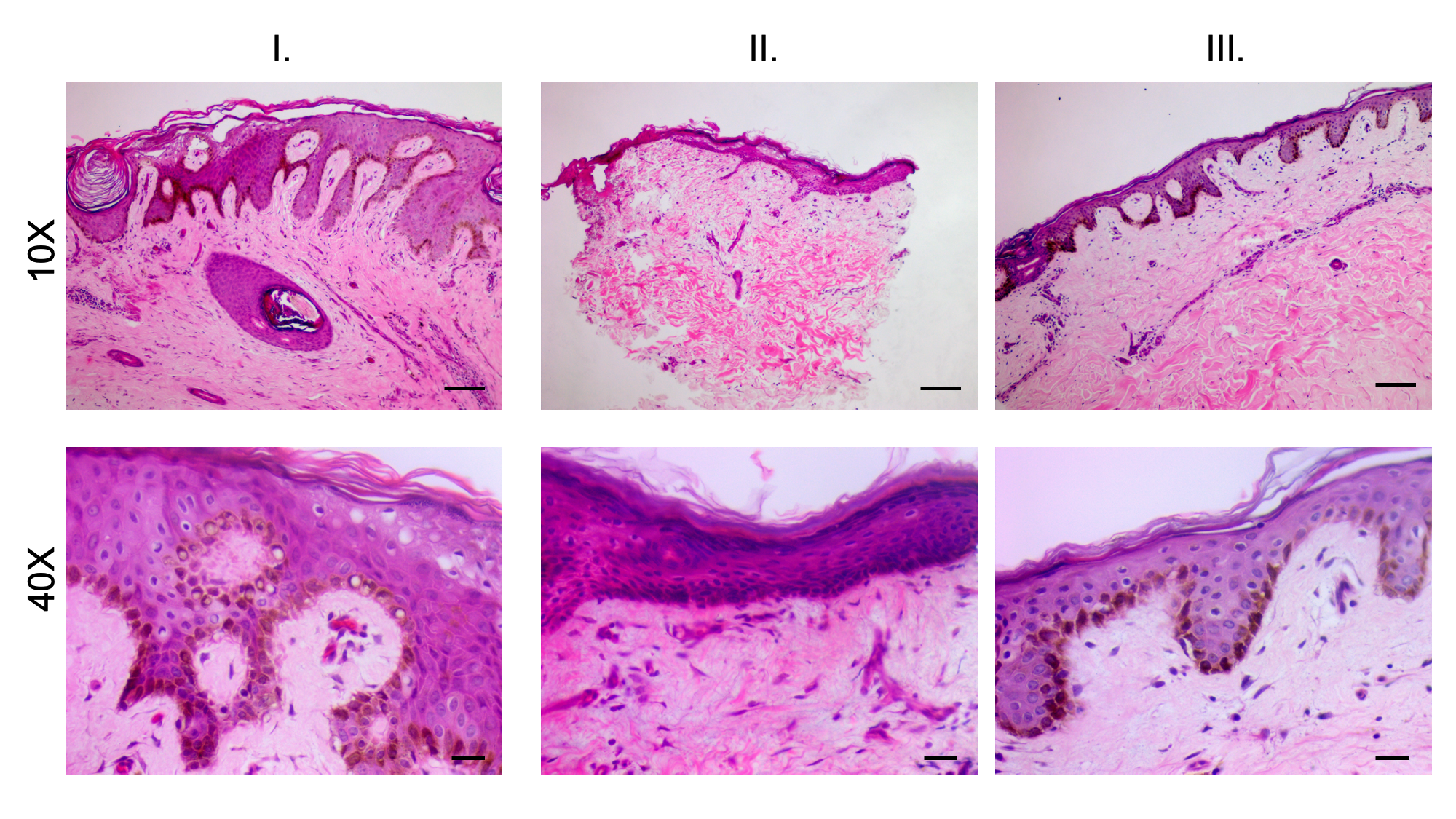

Supplement: S9 Fig — Melanocytes were cultured from biopsies regardless of pigmentation phenotype. Punch biopsies of distinct regions of hyper- (I), hypo- (II), and normally-pigmented (III) scar and skin were taken and were FFPE and H&E stained. Images are from Subject #10 in Table 2. (Scale bar = 100 μm for 10X, and 20 μm for 40X). (TIF) [file pone.0248985.s009.tif]

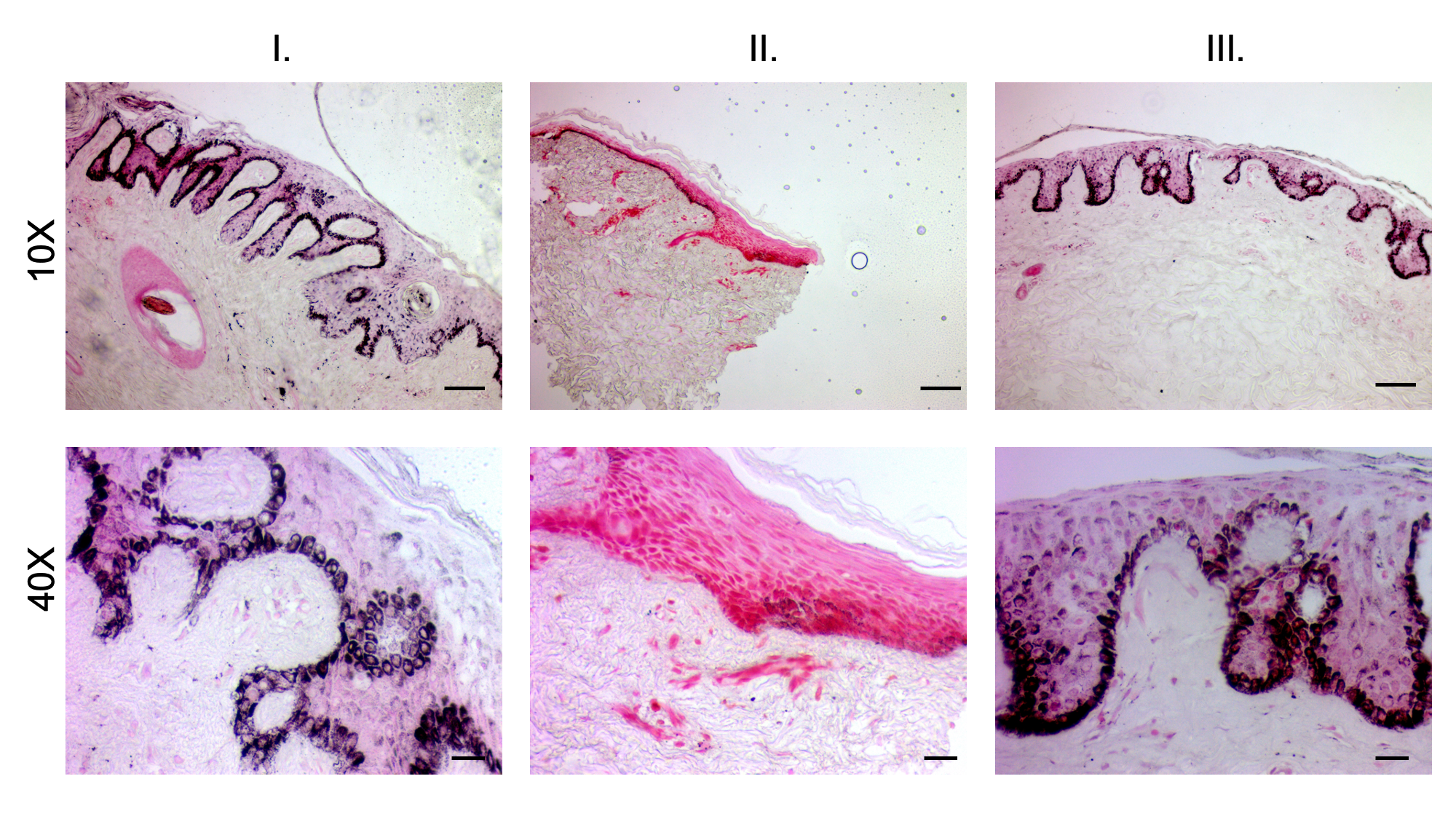

Supplement: S10 Fig — Melanocytes were cultured from biopsies regardless of pigmentation phenotype. Punch biopsies of distinct regions of hyper- (I), hypo- (II), and normally-pigmented (III) scar and skin were taken and were FFPE and Fontana-Masson stained (B). Images are from Subject #10 in Table 2. (Scale bar = 100 μm for 10X, and 20 μm for 40X). (TIF) [file pone.0248985.s010.tif]

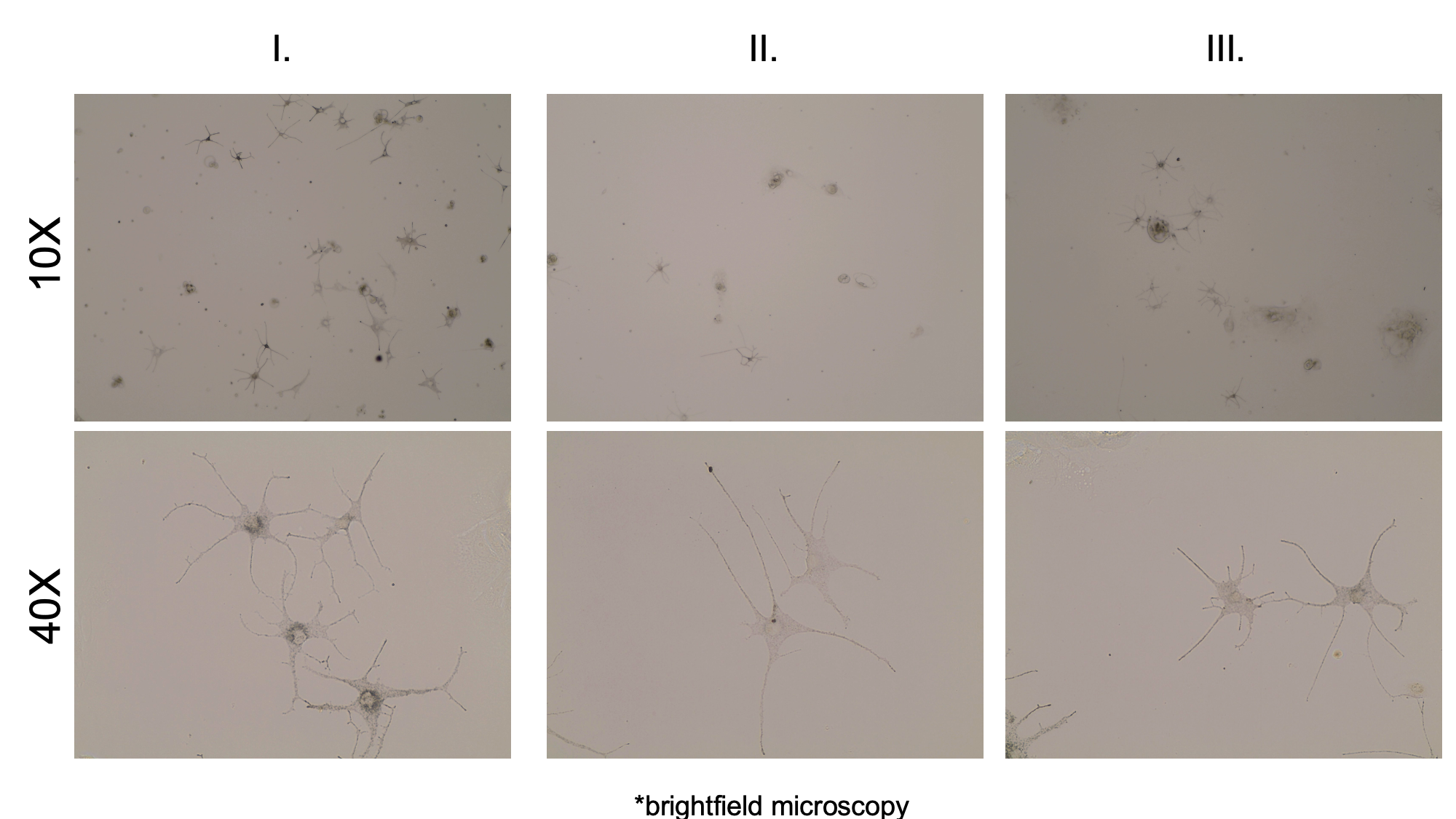

Supplement: S11 Fig — Melanocytes were cultured from biopsies regardless of pigmentation phenotype. Punch biopsies of distinct regions of hyper- (I), hypo- (II), and normally-pigmented (III) scar and skin were taken and treated with dispase to isolate epidermal cells which were seeded in culture. Images were taken under bright field microscopy at a fixed light intensity after 3 days in culture. Images are from Subject #10 in Table 2. (Scale bar = 100 μm for 10X, and 20 μm for 40X). (TIF) [file pone.0248985.s011.tif]

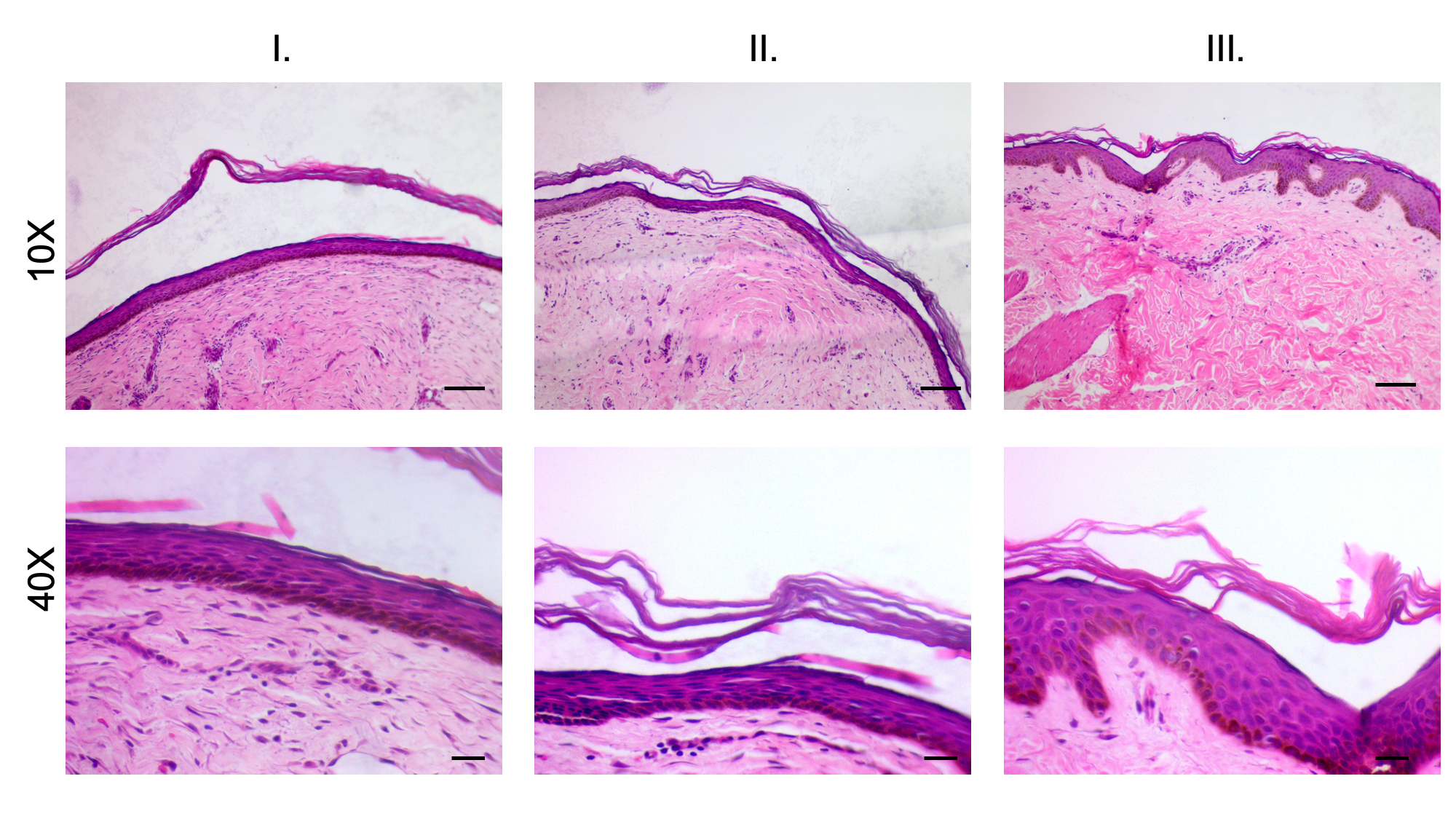

Supplement: S12 Fig — Melanocytes were cultured from biopsies regardless of pigmentation phenotype. Punch biopsies of distinct regions of hyper- (I), hypo- (II), and normally-pigmented (III) scar and skin were taken and were FFPE and H&E stained. Images are from Subject #11 in Table 2. (Scale bar = 100 μm for 10X, and 20 μm for 40X). (TIF) [file pone.0248985.s012.tif]

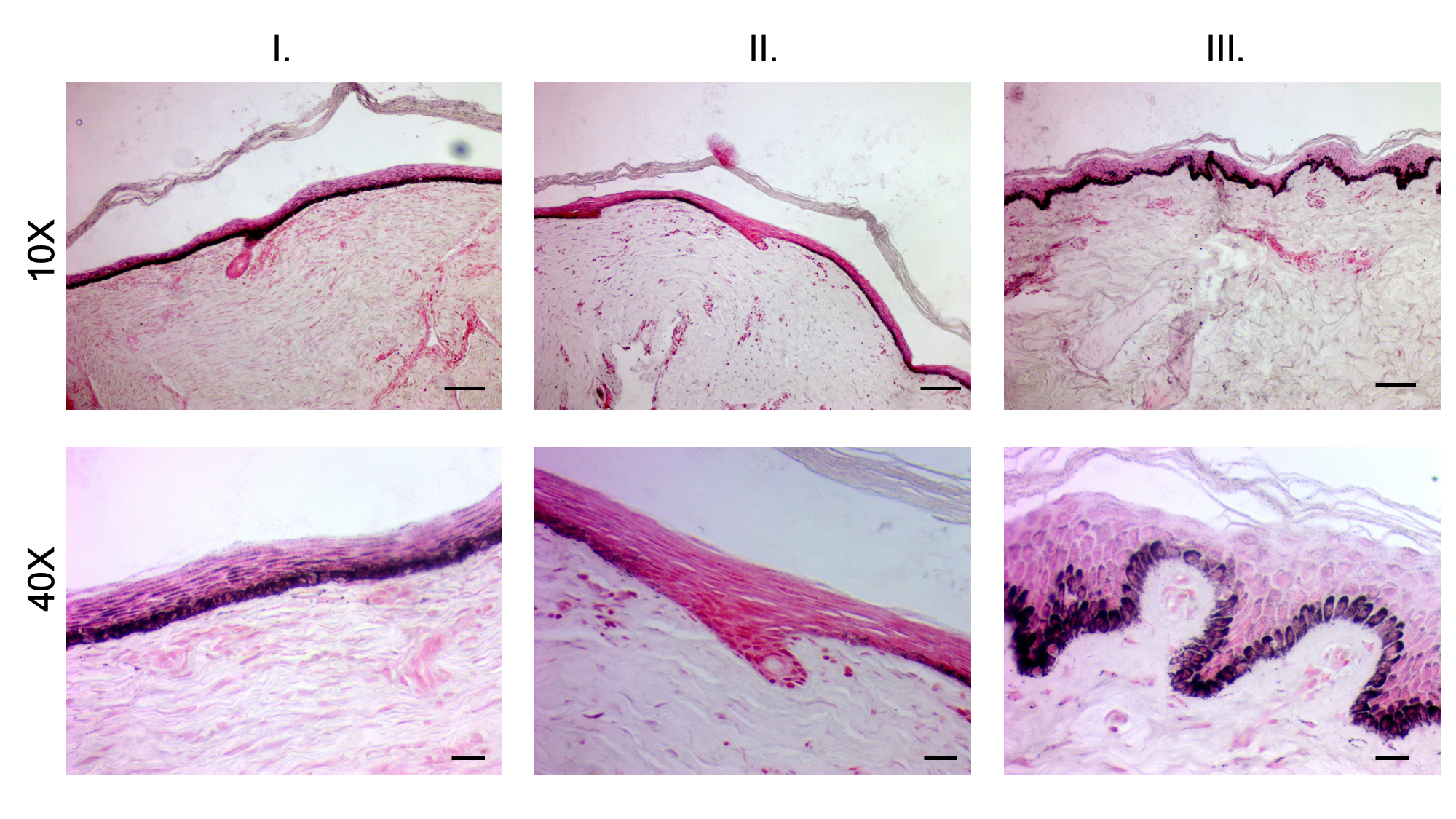

Supplement: S13 Fig — Melanocytes were cultured from biopsies regardless of pigmentation phenotype. Punch biopsies of distinct regions of hyper- (I), hypo- (II), and normally-pigmented (III) scar and skin were taken and were FFPE and Fontana-Masson stained (B). Images are from Subject #11 in Table 2. (Scale bar = 100 μm for 10X, and 20 μm for 40X). (TIF) [file pone.0248985.s013.tif]

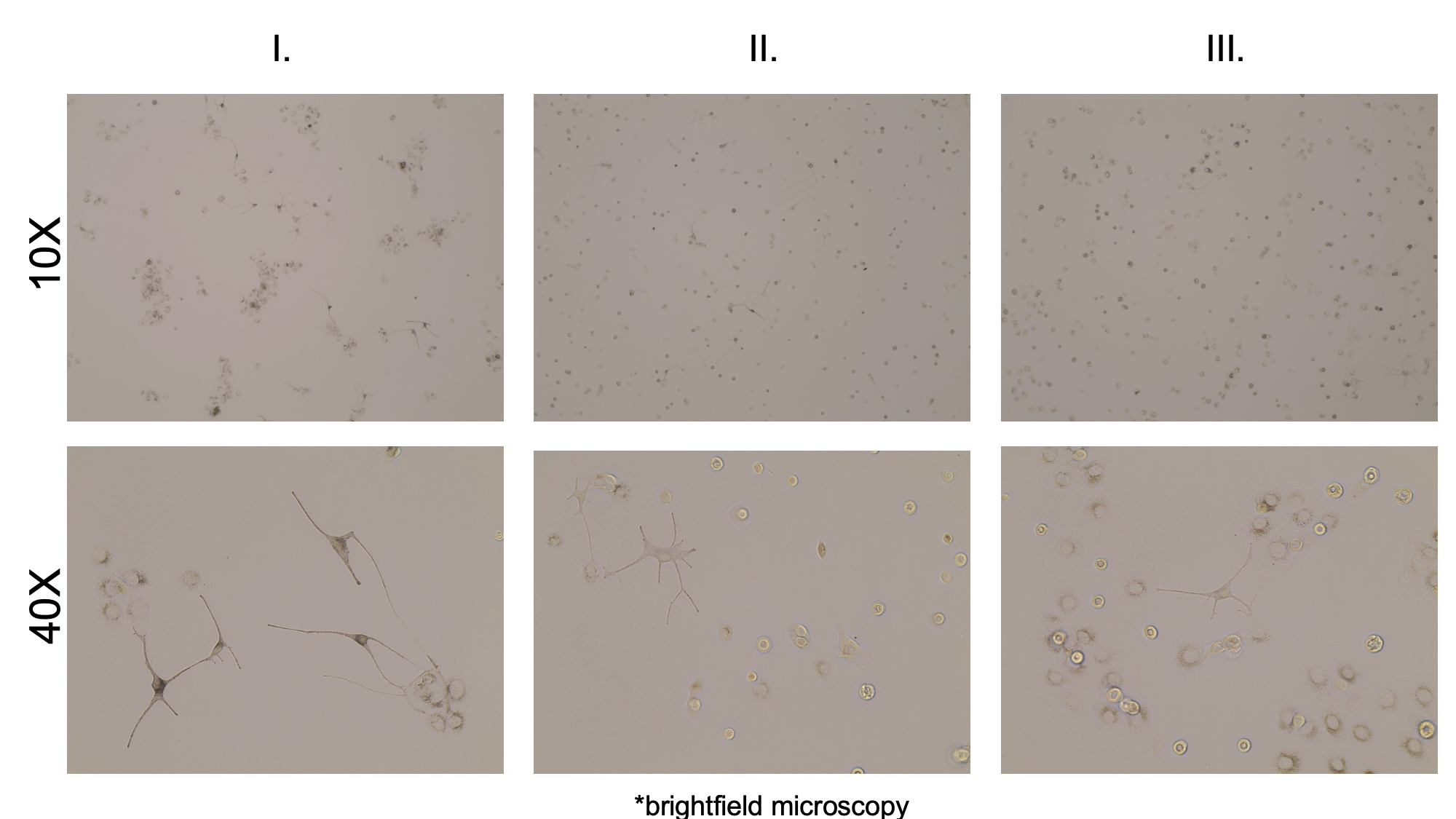

Supplement: S14 Fig — Melanocytes were cultured from biopsies regardless of pigmentation phenotype. Punch biopsies of distinct regions of hyper- (I), hypo- (II), and normally-pigmented (III) scar and skin were taken and treated with dispase to isolate epidermal cells which were seeded in culture. Images were taken under bright field microscopy at a fixed light intensity after 3 days in culture. Images are from Subject #11 in Table 2. (Scale bar = 100 μm for 10X, and 20 μm for 40X). (TIF) [file pone.0248985.s014.tif]

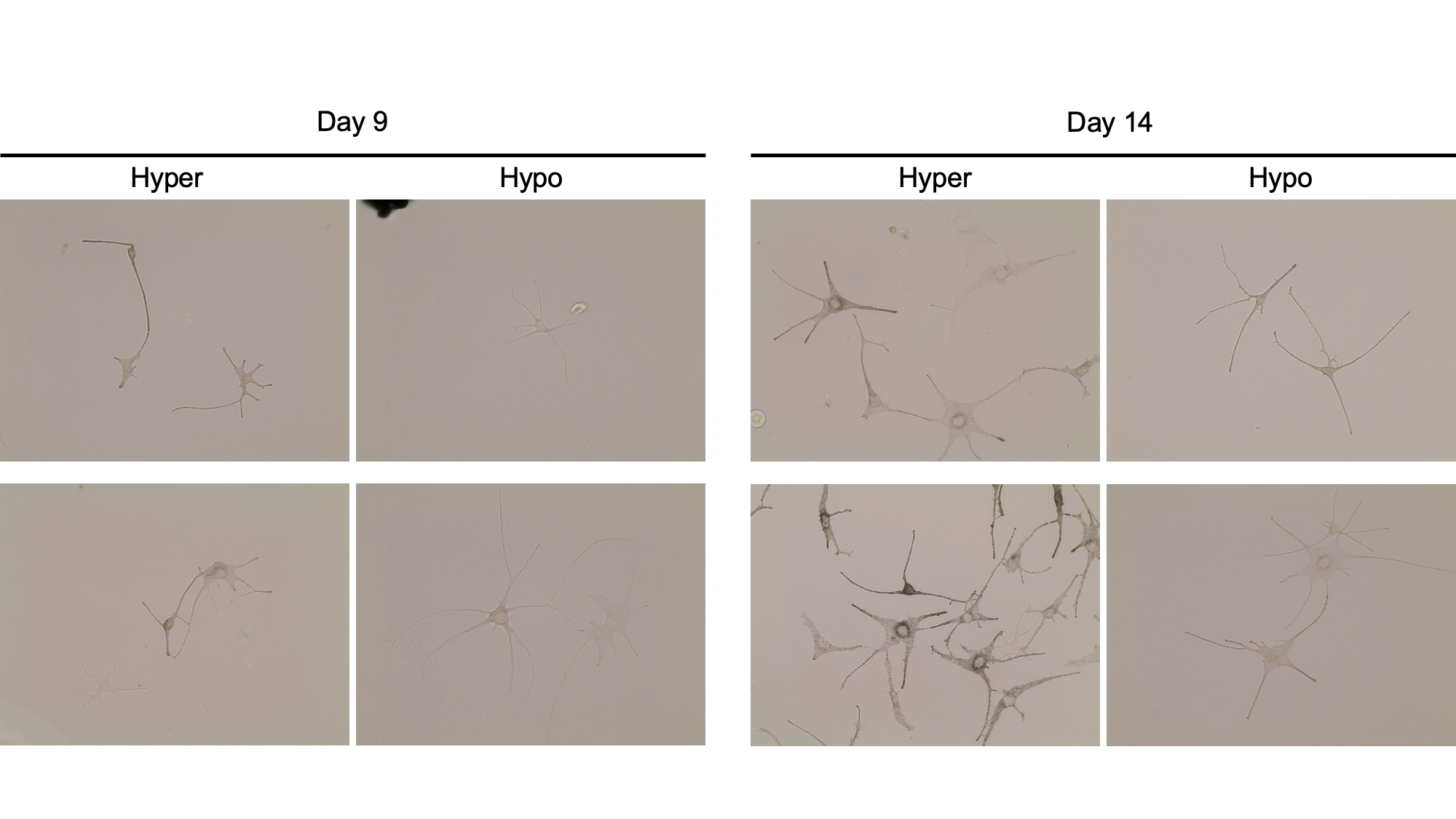

Supplement: S15 Fig — Punch biopsies of distinct regions of hyper- and hypo-pigmented scar were taken during a pre-planned surgical excision and were treated with dispase to isolate epidermal cells which were seeded in culture in media containing α-MSH. Images were taken under bright field microscopy at a fixed light intensity at Days 9 and 14. (TIF) [file pone.0248985.s015.tif]

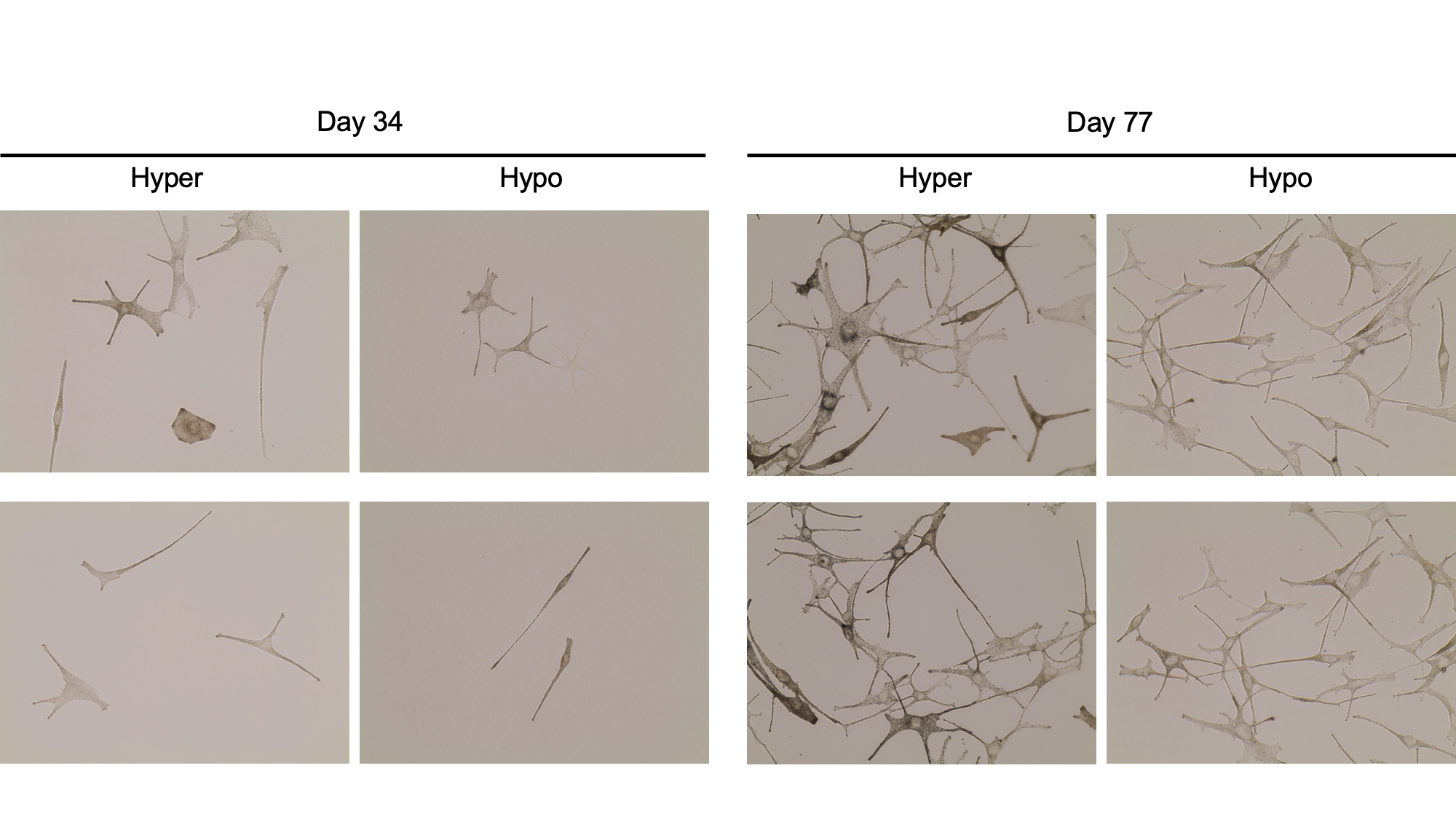

Supplement: S16 Fig — Punch biopsies of distinct regions of hyper- and hypo-pigmented scar were taken during a pre-planned surgical excision and were treated with dispase to isolate epidermal cells which were seeded in culture in media containing α-MSH. Images were taken under bright field microscopy at a fixed light intensity at Days 34 and 77. (TIF) [file pone.0248985.s016.tif]
