# Supplementary material for: Hypopigmented burn hypertrophic scar contains melanocytes that can be signaled to re-pigment by synthetic alpha-melanocyte stimulating hormone in vitro
Source: PLoS One. 2021 Mar 25;16(3):e0248985. doi: 10.1371/journal.pone.0248985 (PMC7993611; doi:10.1371/journal.pone.0248985)
Supplement: Attachment — Submitted filename: Response to Decision Letter_09FEB2021.pdf [file pone.0248985.s017.pdf]

MedStar Health Research Institute

Firefighters' Burn and Surgical Research Laboratory

108 Irving Street, NW
Washington, DC 20010
202.877.2086 PHONE
202.877.2012 FAX
medstarresearch.org

Burn and Surgery Research
MedStar Washington Hospital Center

February 9, 2021

Dr. Andrew Taylor

Academic Editor

PLOS One

Dear Dr. Taylor,

Thank you for the opportunity to provide a response to reviewer comments and edits to the manuscript. We believe we have adequately addressed each comment. None of the material has been published or is under consideration for publication elsewhere. Every author listed meets the qualifications for authorship and has had the opportunity to read and comment upon the re-submitted manuscript. We look forward to hearing back about your decision. Point-by-point response to reviewers are listed below and responses are in **bold** and sections that were added to the manuscript are **highlighted and bolded**:

Additional Editor Comments:

The manuscript is well presented with some important information missing. Besides, the issues raised by the reviewer, there does need to be in the methods section a paragraph on statistical analysis. This should describe all the statistical tests that were conducted in the manuscript and which data sets were tested. In addition in figure 14C, there is no indication of statistically relevant up-regulation of TYR, TYRP1, and DCT. Are the values greater than 1?

We thank you for your thoughtful review. We have added a statistical analysis section to the methods. We have adjusted Figure 14C and have explained the analysis as indicated below.

Journal Requirements:

When submitting your revision, we need you to address these additional requirements.

1) Please ensure that your manuscript meets PLOS ONE's style requirements, including those for file naming.

We have revised our style requirements to be in line with the provided templates.

2) As part of your revisions, please address the following with respect to the pig study: (1) a brief description about monitoring parameters, humane endpoints and any unanticipated adverse events that occurred ; and (2) sample size justification. Thank you for your attention in this matter.

We have added the following details to the methods section under "Duroc Pig Model":

"Sample sizes were reflective of those needed to acquire pilot data, and no power calculation was performed. During all animal procedures, heart rate and oxygen saturation were monitored during induction. During the procedures, heart rate, blood pressure, respiratory rate, end tidal CO_2, oxygen saturation, and temperature were monitored and adjusted to ensure appropriate planes of anesthesia. On the wound creation days, warmed intravenous maintenance fluids were administered."

"At the final timepoint, animals were given an intravenous dose of 4.2M potassium chloride solution at 2 mmol/kg for euthanasia and death was confirmed with flatline vital signs. There were no unanticipated adverse events during these procedures."

3) Thank you for stating in your Funding Statement:

We have updated our funding statement to:

"This research was funded in part by the Georgetown University Medical Center Student Research Grant Program. The authors declare no conflicts of interest. There was no additional external funding received for this study."

4) Please include captions for your Supporting Information files at the end of your manuscript, and update any in-text citations to match accordingly. Please see our Supporting Information guidelines for more information: http://journals.plos.org/plosone/s/supporting-information.

We have updated the supporting information files to comply with the style guidelines.

Reviewer #1: This is an interesting manuscript but needs some additional clarifications

1. Figure 2 - significant difference - i could not see what test was used - for this or any other significant finding. It would be clearer if stats approach was clearly outlined in the methods as a single section.

We have added a statistical analysis section to the methods.

2. Re Figure 3 - states in text lines 168-172 that this shows key features of HTS. none of these features are highlighted, or measured, or quantified in the manuscript and the images do not show any of these features clearly at all.

You are right, Figure 3 does not do an adequate job of displaying these features. We have added Supplemental Figure 1 in order to show these differences more clearly. We have also added this sentence to the manuscript:

“These features of scar have been quantitatively measured in the same pig samples in a recent report where we showed statistical increases in the above mentioned parameters [12].”

In addition, we now reference our recently published paper which quantitatively measures these features of scar in the pig samples which we refer. The figure to which we refer is replicated below for your reference.

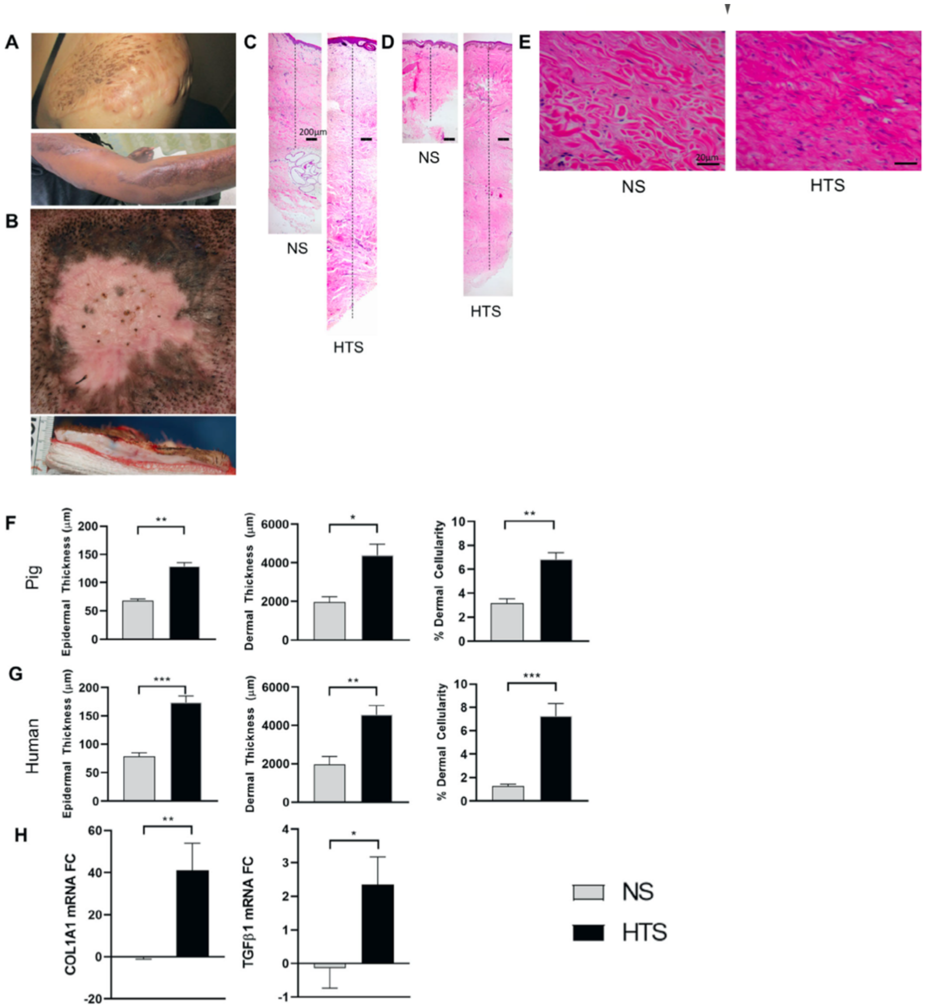

FIGURE 1 Macroscopic, histologic, and molecular appearance of hypertrophic scar. Photos of patients with hypertrophic scarring, A. Photo of duroc pig HTS created by excisional wounding, and excised HTS showing thickness of ~5 mm, B.H&E stains of porcine NS (left) and HTS (right), Scale bar = 200 μm, dotted line = dermal thickness, C. H&E stains of human NS (left) and HTS (right), Scale bar = 200 μm, dotted line = dermal thickness, D. High-magnification images of H&E-stained reticular dermis of human NS (left) and HTS (right), demonstrating dense collagen and hypercellularity of HTS dermis, Scale bar = 20 μm, E. Epidermal thickness, dermal thickness, dermal cellularity, and dermal blood vessel density of porcine NS and HTS; average ± SEM of n = 3 HTS and n = 3 NS biopsies is presented for each, F. Epidermal thickness, dermal thickness, dermal cellularity, and dermal blood vessel density of human NS and HTS; average ± SEM of n = 14 HTS and n = 4 NS biopsies for thickness and cellularity measurements; n = 5 HTS and n = 4 NS biopsies for blood vessel density measurement, G. Human HTS gene expression of COL1A1 (left) and TGFβ1 (right) determined by qRT-PCR $\Delta\Delta C_t$ analysis using GAPDH as the housekeeping control gene. Average mRNA expression fold changes ± SEM of n = 16 HTS and n = 7 NS samples is presented, H. FC, fold change. *P <.05, **P <.01, ***P <.001 [Color figure can be viewed at wileyonlinelibrary.com]

3. The figure legend also states that normal skin was taken but these are not shown only 3 different scar regions.

In Figure 3, letter C is normal skin.

4. Similarly for figure 7 there is no normal skin section but this is stated in figure legend.

In Figure 7, letter C is normal skin.

5. Re Fig 14 and upregulation of genes - was this significantly increased. how was it analysed (stats)?

When conducting the qRT-PCR, each patient was normalized to their own control. Therefore, each control is inherently=1. Therefore, a statistical comparison between the treated and the controls is not appropriate, because we would be comparing each patient control (fold change=1 for all patients) to the fold changes in the treated groups. Therefore, we have added additional language to the results section and statistics section to further explain this point.

"Each patient was normalized to their own control levels of gene expression without treatment. Average levels of fold change above a cut-off=1.5 were accepted as gene up-regulation when analyzing data, however, a statistical comparison was not done due to the variable transcript expression among different patients which precluded using a single control."

"When analyzing the qRT-PCR data, a fold change cut-off>1.5 was set, but a statistical comparison was not performed because each patient was normalized to their own control levels of gene expression without treatment."

6. The discussion is good and it is useful that approaches that were unsuccessful are also described in this section re culturing.

   We appreciate the positive feedback.

7. References are not formatted and have additional pieces of text - this might be easily fixed with endnote or similar bibliography manager?

   We have adjusted all references to conform to PLOS one's style guidelines.

On behalf of the authors,

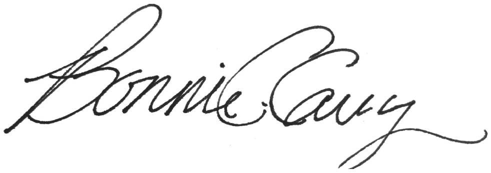

Bonnie C. Carney, PhD

bonnie.c.carney@medstar.net

202-877-2962
