# Supplementary material for: Hypopigmented burn hypertrophic scar contains melanocytes that can be signaled to re-pigment by synthetic alpha-melanocyte stimulating hormone in vitro
Source: PLoS One. 2021 Mar 25;16(3):e0248985. doi: 10.1371/journal.pone.0248985 (PMC7993611; doi:10.1371/journal.pone.0248985)
Supplement: Peer review history

# Peer review: Decision Letter 0

**DOI:** 10.1371/journal.pone.0248985.r001

**Authors:** Andrew W Taylor

**Transfer Alert**

This paper was transferred from another journal. As a result, its full editorial history (including decision letters, peer reviews and author responses) may not be present.

8 Feb 2021

PONE-D-20-39366

Hypopigmented burn hypertrophic scar contains melanocytes that can be signaled to re-pigment by synthetic alpha-melanocyte stimulating hormone in vitro

PLOS ONE

Dear Dr. Carney,

Thank you for submitting your manuscript to PLOS ONE. After careful consideration, we feel that it has merit but does not fully meet PLOS ONE’s publication criteria as it currently stands. Therefore, we invite you to submit a revised version of the manuscript that addresses the points raised during the review process.

Please submit your revised manuscript by Mar 25 2021 11:59PM. If you will need more time than this to complete your revisions, please reply to this message or contact the journal office at plosone@plos.org. When you're ready to submit your revision, log on to https://www.editorialmanager.com/pone/ and select the 'Submissions Needing Revision' folder to locate your manuscript file.

Please include the following items when submitting your revised manuscript:

- A rebuttal letter that responds to each point raised by the academic editor and reviewer(s). You should upload this letter as a separate file labeled 'Response to Reviewers'.
- A marked-up copy of your manuscript that highlights changes made to the original version. You should upload this as a separate file labeled 'Revised Manuscript with Track Changes'.
- An unmarked version of your revised paper without tracked changes. You should upload this as a separate file labeled 'Manuscript'.

If you would like to make changes to your financial disclosure, please include your updated statement in your cover letter. Guidelines for resubmitting your figure files are available below the reviewer comments at the end of this letter.

If applicable, we recommend that you deposit your laboratory protocols in protocols.io to enhance the reproducibility of your results. Protocols.io assigns your protocol its own identifier (DOI) so that it can be cited independently in the future. For instructions see: http://journals.plos.org/plosone/s/submission-guidelines#loc-laboratory-protocols

We look forward to receiving your revised manuscript.

Kind regards,

Andrew W Taylor, Ph.D.

Academic Editor

PLOS ONE

Additional Editor Comments:

The manuscript is well presented with some important information missing. Besides, the issues raised by the reviewer, there does need to be in the methods section a paragraph on statistical analysis. This should describe all the statistical tests that were conducted in the manuscript and which data sets were tested. In addition in figure 14C, there is no indication of statistically relevant up-regulation of TYR, TYRP1, and DCT. Are the values greater than 1?

Journal Requirements:

When submitting your revision, we need you to address these additional requirements.

1) Please ensure that your manuscript meets PLOS ONE's style requirements, including those for file naming. The PLOS ONE style templates can be found at

https://journals.plos.org/plosone/s/file?id=wjVg/PLOSOne_formatting_sample_main_body.pdf and

https://journals.plos.org/plosone/s/file?id=ba62/PLOSOne_formatting_sample_title_authors_affiliations.pdf

2) As part of your revisions, please address the following with respect to the pig study: (1) a brief description about monitoring parameters, humane endpoints and any unanticipated adverse events that occurred ; and (2) sample size justification. Thank you for your attention in this matter.

3) Thank you for stating in your Funding Statement:

 [This research was funded in part by the Georgetown University Medical Center

Student Research Grant Program.]. 

Please provide an amended statement that declares *all* the funding or sources of support (whether external or internal to your organization) received during this study, as detailed online in our guide for authors at http://journals.plos.org/plosone/s/submit-now.  Please also include the statement “There was no additional external funding received for this study.” in your updated Funding Statement.

Please include your amended Funding Statement within your cover letter. We will change the online submission form on your behalf.

4) Please include captions for your Supporting Information files at the end of your manuscript, and update any in-text citations to match accordingly. Please see our Supporting Information guidelines for more information: http://journals.plos.org/plosone/s/supporting-information.

[Note: HTML markup is below. Please do not edit.]

Reviewers' comments:

Reviewer's Responses to Questions

**Comments to the Author**

1. Is the manuscript technically sound, and do the data support the conclusions?

The manuscript must describe a technically sound piece of scientific research with data that supports the conclusions. Experiments must have been conducted rigorously, with appropriate controls, replication, and sample sizes. The conclusions must be drawn appropriately based on the data presented. 

Reviewer #1: Yes

**********

2. Has the statistical analysis been performed appropriately and rigorously? 

Reviewer #1: I Don't Know

**********

3. Have the authors made all data underlying the findings in their manuscript fully available?

The PLOS Data policy requires authors to make all data underlying the findings described in their manuscript fully available without restriction, with rare exception (please refer to the Data Availability Statement in the manuscript PDF file). The data should be provided as part of the manuscript or its supporting information, or deposited to a public repository. For example, in addition to summary statistics, the data points behind means, medians and variance measures should be available. If there are restrictions on publicly sharing data—e.g. participant privacy or use of data from a third party—those must be specified.

Reviewer #1: Yes

**********

4. Is the manuscript presented in an intelligible fashion and written in standard English?

PLOS ONE does not copyedit accepted manuscripts, so the language in submitted articles must be clear, correct, and unambiguous. Any typographical or grammatical errors should be corrected at revision, so please note any specific errors here.

Reviewer #1: Yes

**********

5. Review Comments to the Author

Please use the space provided to explain your answers to the questions above. You may also include additional comments for the author, including concerns about dual publication, research ethics, or publication ethics. (Please upload your review as an attachment if it exceeds 20,000 characters)

Reviewer #1: This is an interesting manuscript but needs some additional clarifications.

Figure 2 - significant difference - i could not see what test was used - for this or any other significant finding. It would be clearer if stats approach was clearly outlined in the methods as a single section.

Re Figure 3 - states in text lines 168-172 that this shows key features of HTS. none of these features are highlighted, or measured, or quantified in the manuscript and the images do not show any of these features clearly at all. The figure legend also states that normal skin was taken but these are not shown only 3 different scar regions. Similarly for figure 7 there is no normal skin section but this is stated in figure legend.

Re Fig 14 and upregulation of genes - was this significantly increased. how was it analysed (stats)?

the discussion is good and it is useful that approaches that were unsuccessful are also described in this section re culturing.

references are not formatted and have additional pieces of text - this might be easily fixed with endnote or similar bibliography manager?

**********

6. PLOS authors have the option to publish the peer review history of their article (what does this mean?). If published, this will include your full peer review and any attached files.

If you choose “no”, your identity will remain anonymous but your review may still be made public.

**Do you want your identity to be public for this peer review?** For information about this choice, including consent withdrawal, please see our Privacy Policy.

Reviewer #1: No

[NOTE: If reviewer comments were submitted as an attachment file, they will be attached to this email and accessible via the submission site. Please log into your account, locate the manuscript record, and check for the action link "View Attachments". If this link does not appear, there are no attachment files.]

While revising your submission, please upload your figure files to the Preflight Analysis and Conversion Engine (PACE) digital diagnostic tool, https://pacev2.apexcovantage.com/. PACE helps ensure that figures meet PLOS requirements. To use PACE, you must first register as a user. Registration is free. Then, login and navigate to the UPLOAD tab, where you will find detailed instructions on how to use the tool. If you encounter any issues or have any questions when using PACE, please email PLOS at figures@plos.org. Please note that Supporting Information files do not need this step.

# Author comment: Author response to Decision Letter 0

**DOI:** 10.1371/journal.pone.0248985.r002

10 Feb 2021

February 9, 2021

Dr. Andrew Taylor 

Academic Editor

PLOS One

Dear Dr. Taylor,

Thank you for the opportunity to provide a response to reviewer comments and edits to the manuscript. We believe we have adequately addressed each comment. None of the material has been published or is under consideration for publication elsewhere. Every author listed meets the qualifications for authorship and has had the opportunity to read and comment upon the re-submitted manuscript. We look forward to hearing back about your decision. Point-by-point response to reviewers are listed below and responses are in bold and sections that were added to the manuscript are highlighted and bolded:

Additional Editor Comments:

The manuscript is well presented with some important information missing. Besides, the issues raised by the reviewer, there does need to be in the methods section a paragraph on statistical analysis. This should describe all the statistical tests that were conducted in the manuscript and which data sets were tested. In addition in figure 14C, there is no indication of statistically relevant up-regulation of TYR, TYRP1, and DCT. Are the values greater than 1?

We thank you for your thoughtful review. We have added a statistical analysis section to the methods. We have adjusted Figure 14C and have explained the analysis as indicated below.

Journal Requirements:

When submitting your revision, we need you to address these additional requirements.

1) Please ensure that your manuscript meets PLOS ONE's style requirements, including those for file naming. 

We have revised our style requirements to be in line with the provided templates. 

2) As part of your revisions, please address the following with respect to the pig study: (1) a brief description about monitoring parameters, humane endpoints and any unanticipated adverse events that occurred ; and (2) sample size justification. Thank you for your attention in this matter.

We have added the following details to the methods section under “Duroc Pig Model”:

“Sample sizes were reflective of those needed to acquire pilot data, and no power calculation was performed. During all animal procedures, heart rate and oxygen saturation were monitored during induction. During the procedures, heart rate, blood pressure, respiratory rate, end tidal CO2, oxygen saturation, and temperature were monitored and adjusted to ensure appropriate planes of anesthesia. On the wound creation days, warmed intravenous maintenance fluids were administered.”

“At the final timepoint, animals were given an intravenous dose of 4.2M potassium chloride solution at 2 mmol/kg for euthanasia and death was confirmed with flatline vital signs. There were no unanticipated adverse events during these procedures.” 

3) Thank you for stating in your Funding Statement:

We have updated our funding statement to:

“This research was funded in part by the Georgetown University Medical Center

Student Research Grant Program. The authors declare no conflicts of interest. There was no additional external funding received for this study.” 

4) Please include captions for your Supporting Information files at the end of your manuscript, and update any in-text citations to match accordingly. Please see our Supporting Information guidelines for more information: http://journals.plos.org/plosone/s/supporting-information.

 We have updated the supporting information files to comply with the style guidelines.

Reviewer #1: This is an interesting manuscript but needs some additional clarifications

1. Figure 2 - significant difference - i could not see what test was used - for this or any other significant finding. It would be clearer if stats approach was clearly outlined in the methods as a single section.

We have added a statistical analysis section to the methods.

2. Re Figure 3 - states in text lines 168-172 that this shows key features of HTS. none of these features are highlighted, or measured, or quantified in the manuscript and the images do not show any of these features clearly at all. 

You are right, Figure 3 does not do an adequate job of displaying these features. We have added Supplemental Figure 1 in order to show these differences more clearly. We have also added this sentence to the manuscript:

“These features of scar have been quantitatively measured in the same pig samples in a recent report where we showed statistical increases in the above mentioned parameters [12].” 

In addition, we now reference our recently published paper which quantitatively measures these features of scar in the pig samples which we refer. The figure to which we refer is replicated below for your reference. 

3. The figure legend also states that normal skin was taken but these are not shown only 3 different scar regions.

In Figure 3, letter C is normal skin. 

4. Similarly for figure 7 there is no normal skin section but this is stated in figure legend.

In Figure 7, letter C is normal skin.

5. Re Fig 14 and upregulation of genes - was this significantly increased. how was it analysed (stats)?

When conducting the qRT-PCR, each patient was normalized to their own control. Therefore, each control is inherently=1. Therefore, a statistical comparison between the treated and the controls is not appropriate, because we would be comparing each patient control (fold change=1 for all patients) to the fold changes in the treated groups. Therefore, we have added additional language to the results section and statistics section to further explain this point. 

“Each patient was normalized to their own control levels of gene expression without treatment. Average levels of fold change above a cut-off=1.5 were accepted as gene up-regulation when analyzing data, however, a statistical comparison was not done due to the variable transcript expression among different patients which precluded using a single control.”

“When analyzing the qRT-PCR data, a fold change cut-off>1.5 was set, but a statistical comparison was not performed because each patient was normalized to their own control levels of gene expression without treatment.”

6. The discussion is good and it is useful that approaches that were unsuccessful are also described in this section re culturing.

We appreciate the positive feedback.

7. References are not formatted and have additional pieces of text - this might be easily fixed with endnote or similar bibliography manager?

We have adjusted all references to conform to PLOS one’s style guidelines.

On behalf of the authors,

Bonnie C. Carney, PhD

bonnie.c.carney@medstar.net

202-877-2962

Attachment Submitted filename: Response to Decision Letter_09FEB2021.pdf Click here for additional data file.

# Editor report: Decision Letter 1

**DOI:** 10.1371/journal.pone.0248985.r003

**Authors:** Andrew W Taylor

10 Mar 2021

Hypopigmented burn hypertrophic scar contains melanocytes that can be signaled to re-pigment by synthetic alpha-melanocyte stimulating hormone in vitro

PONE-D-20-39366R1

Dear Dr. Carney,

We’re pleased to inform you that your manuscript has been judged scientifically suitable for publication and will be formally accepted for publication once it meets all outstanding technical requirements.

Within one week, you’ll receive an e-mail detailing the required amendments. When these have been addressed, you’ll receive a formal acceptance letter and your manuscript will be scheduled for publication.

An invoice for payment will follow shortly after the formal acceptance. To ensure an efficient process, please log into Editorial Manager at http://www.editorialmanager.com/pone/, click the 'Update My Information' link at the top of the page, and double check that your user information is up-to-date. If you have any billing related questions, please contact our Author Billing department directly at authorbilling@plos.org.

If your institution or institutions have a press office, please notify them about your upcoming paper to help maximize its impact. If they’ll be preparing press materials, please inform our press team as soon as possible -- no later than 48 hours after receiving the formal acceptance. Your manuscript will remain under strict press embargo until 2 pm Eastern Time on the date of publication. For more information, please contact onepress@plos.org.

Kind regards,

Andrew W Taylor, Ph.D.

Academic Editor

PLOS ONE

Additional Editor Comments (optional):

Reviewers' comments:

# Editor report: Acceptance letter

**DOI:** 10.1371/journal.pone.0248985.r004

**Authors:** Andrew W Taylor

16 Mar 2021

PONE-D-20-39366R1 

Hypopigmented burn hypertrophic scar contains melanocytes that can be signaled to re-pigment by synthetic alpha-melanocyte stimulating hormone in *vitro*

Dear Dr. Carney:

I'm pleased to inform you that your manuscript has been deemed suitable for publication in PLOS ONE. Congratulations! Your manuscript is now with our production department. 

If your institution or institutions have a press office, please let them know about your upcoming paper now to help maximize its impact. If they'll be preparing press materials, please inform our press team within the next 48 hours. Your manuscript will remain under strict press embargo until 2 pm Eastern Time on the date of publication. For more information please contact onepress@plos.org.

If we can help with anything else, please email us at plosone@plos.org. 

Thank you for submitting your work to PLOS ONE and supporting open access. 

Kind regards, 

PLOS ONE Editorial Office Staff

on behalf of

Dr. Andrew W Taylor 

Academic Editor

PLOS ONE
